# Supplementary material for: WordCommentsAnalyzer: A windows software tool for qualitative research
Source: F1000Res. 2018 Sep 4;7:536. Originally published 2018 May 3. [Version 2] doi: 10.12688/f1000research.14819.2 (PMC6137411; doi:10.12688/f1000research.14819.2)
Supplement: Supplementary file 3 [file f1000research-7-17614-s0002.tgz › b2a79496-4ada-47c4-9682-a99ce867836d.zip!/Eight Iranian Health Journals/Journal of Knowledge and Health_31.docx]

© Journal of Knowledge and Health. This work is licensed under CC BY 4.0 (https://creativecommons.org/licenses/by/4.0/).

Title: انتقال دانش یک ضرورت برای اپيدميولوژي نوین

http://knh.shmu.ac.ir/index.php/site/article/view/953

توجه به بهره­برداری از دانش یک نیاز برای تحقق جامعه دانش بنیان است. موضوع اصلی این است که تولید علم، به تنهایی ضامن حل مشکلات سلامت جامعه و پیشرفت نیست و بایستی از بهترین شیوه ها برای ایجاد تغییر استفاده نمود. دو شیوه کلی در مداخله­های انتقال دانش مطرح هستند. یکی تقویت ابزارهای مطرح در تصمیم­گیری مبتنی بر شواهد و تولید خروجی­های استاندارد مناسب با گروه­های مخاطب مانند راهنماهای بالینی و بهداشت عمومی و یافته­های سیاست­گذاری و دیگری تغییر در زیرساخت­های تحقیق، روابط تولیدکنندگان و بهره­گیران از شواهد علمی. در این عرصه نقش اپیدمیولوژی به سه شکل مورد توجه می­باشد. اول در شکل عمومی و مانند سایر علوم کاربردی است که باید از شیوه­ها و ابزارهای انتقال دانش برای تقویت بهره­برداری از شواهد استفاده شود. هر چند در این قسم نیز رسالت اپیدمیولوژی در تولید و بومی­سازی این گونه شیوه ها و ابزارهای مبتنی بر شواهد خاص است. در شکل دوم موضوع اصلی انتقال دانش، تغییر رفتار است. تغییر رفتار ارایه­دهندگان خدمات، مردم، بیماران، مدیران و غیره. در این عرصه موضوع فعالیت اپیدمیولوژی، تغییر رفتار این گروه­های مختلف است و رفتارها و یا عوامل موثر بر آن را بایستی با روش­های اپیدمیولوژیک معمول مورد تحلیل قرار داد. بالاخره این که در شکل سوم، انتقال دانش ضامن وجود روش­های اختصاصی اپیدمیولوژیک مانند تحلیل شبکه­های اجتماعی، مدل سازی معادله­های ساختاری (structural equation modeling) و تحلیل انتشار نوآوری­ها است. به نظر می­رسد که آموزش­های انتقال دانش باید در برنامه­های عمومی آموزش روش تحقیق و به­ویژه برای همکاران اپیدمیولوژیست جای گرفته و از موضوعات اصلی برای کسب مهارت­های لازم برای آن­ها باشد.

Title: نابرابری در اختلالات بینایی مردم شاهرود

http://knh.shmu.ac.ir/index.php/site/article/view/962

مقدمه: مطالعه کوهورت چشم پزشکی شاهرود به­منظور تعیین شیوع و بروز بیماری­های اصلی چشم در سنین بالا و مشخص کردن تعیین­کنندگان آن طراحی و فاز اول آن در سال­های 1387 و 1388 اجرا شده است. جامعه هدف در اين مطالعه افراد بين 40 تا 64 سال شهر شاهرود مي­باشند. برحسب وضعیت افراد نسبت به میانه نمره حاصله از وجود انواع دارايی­های ثابت، جامعه مورد بررسی به دو گروه برخوردار و غیربرخوردار گروه­بندی شد، اختلاف میانگین نمره دید دو گروه (بر حسب logMar) با استفاده از روش تجزیه اکساکا (Oaxaca– Blinder decomposition)، تحلیل و عوامل ایجادکننده نابرابری در میزان دید (Presenting vision) مشخص و سهم تأثیر هر کدام روشن شد. یافته ها: از مجموع 5190 شركت­كننده در مطالعه، 5182 نفر معاینه اپتومتري شده كه 342 نفر (6/6%) در چشم بهتر دید بدتر از 3/0 در مقیاس لگمار (بدتر از 40/20) داشتند. این نسبت در گروه برخوردار 23/4% و در گروه غیربرخوردار 58/9% بود. این اختلاف از نظر آماری معنادار می­باشد (0001/0P<). 57% از اختلاف بین میانگین دید فرد در دو گروه مربوط به تغييرات متغیرهایی چون سطح سواد فرد، شغل، وضعیت مسکن، سن، جنس و سطح سواد سرپرست خانواده و 43% مربوط به تفاوت نحوه تأثیر این متغیرها ناشی از اختلاف عرض از مبدا و ضرایب آن­ها در دو گروه بود. قسمت اعظم اختلاف میانگین دو گروه ناشی از سن، وضعیت تأهل و میزان تحصیلات سرپرست خانواده بود. نتیجه ­گیری: نابرابری در اختلالات بینایی قابل توجه و علاوه بر عوامل شناخته شده در ایجاد این اختلالات، عوامل اقتصادی- اجتماعی هم نقش قابل توجه­ای دارند. برای موفقیت برنامه­های پیشگیری و درمان باید به این عوامل توجه كرده و مداخله­های مناسب طراحی نمود.

Title: اپیدمیولوژی آلودگی هوا، روند مطالعات و چالش‌های پیش رو

http://knh.shmu.ac.ir/index.php/site/article/view/966

مقدمه: مطالعات زیادی طی چند دهه اخیر در خصوص اثرات آلودگی هوا بر سلامت انسان انجام شده­اند ولی هیچ یک تا کنون نتوانسته­اند به­طور جامع و مناسبی به بررسی کلیه اثرات آن بپردازند. در این مقاله قصد داریم با بررسی نمونه­ای از مطالعات انجام شده در خصوص اثرات آلودگی هوا بر سلامت در 19 سال گذشته، روند مطالعات و مشکلات آن­ها را بررسی نماییم. مواد و روش­ها: با استفاده از جستجوی کلید واژه­های مرتبط با آلاینده­های هوا و اثرات آن­ها بر سلامت در بانک (ISI) Web of Science طی سال­های 1990 تا 2009 مقالات مرتبط استخراج شد و با مطالعه نمونه تصادفی مشتمل بر 50 مقاله از هر سال و مطالعه چکیده مقالات، نوع مقاله (متدولوژی مورد استفاده) و یا حیطه آن (بررسی مواجهه، ارتباط مواجهه با پی­آمد، بررسی در محیط­های باز یا بسته و یا بحث­های متدولوژیک و آماری) مشخص گردیده و روند آن­ها طی زمان بررسی گردید. در خاتمه با جمع­بندی مطالعاتی که اخیراً در خصوص روش­شناسی اپیدمیولوژی آلودگی هوا منتشر شده‌اند، چالش­های این نوع مطالعات مورد بحث قرار گرفته‌اند. يافته‌ها: بیش از 5700 مقاله که طی مدت 19 سال (از 1991 تا 2009) منتشر شده بودند حاصل شد. مطالعات مروری حجم قابل ملاحظه­ای از مقالات منتشر شده را به خود اختصاص داده بودند. با گذشت زمان سهم مطالعات مروری کاهش یافته و نسبت مطالعاتی که به موضوعات متدولوژی و به خصوص بررسی مواجهه پرداخته بودند افزایش یافته است. در مجموع، چه در مطالعات قدیمی و چه در مطالعات نسبتاً جدیدتر سهم ناچیزی به بررسی اثرات آلاینده­های فضاهای بسته (indoor) اختصاص یافته است. روش­های نسبتاً جدیدتر نظیر case-crossover نیز کمابیش بین مطالعات منتشر شده مشاهده می­شوند. چالش اصلی که در زمینه اپیدمیولوژی آلودگی هوا وجود دارد این است که هیچ نوع مطالعه­ای نمی­تواند به همه جنبه­های اثرات آلودگی هوا بر سلامت انسان به درستی بپردازد: مطالعات قدیمی­تر از الگوی بیماری­های واگیردار برای این منظور استفاده کرده­اند. دراین قبیل مطالعات تنها اثرات افزایش­های ناگهانی و بسیار شدید آلودگی هوا قابل بررسی است. در نسل بعدی از مقایسه­های بین مکانی استفاده شده که گرچه توانایی بررسی اثرات مزمن مواجهه با آلودگی هوا را دارد و ماحصل آن بسیاری از استانداردهای آلاینده­های هواست ولی به شدت تحت تأثیر اثرات متغیرهای مخدوش­کننده متعدد فردی و محیطی قرار دارد. باید توجه داشت اهمیت آلودگی هوا آن است که بر بخش بسیار بزرگی از جامعه تأثير دارد، لذا یک خطر نسبی خیلی کوچک (در حد 02/1) می­تواند تعداد زیادی پی­آمد اضافی به همراه داشته باشد. از طرف دیگر این ارتباط نسبتاً کوچک، به سادگی می­تواند تحت تأثیر مخدوش­کننده­ها ایجاد و یا پوشانده شود. نسل سوم مطالعات اپیدمیولوژی آلودگی هوا به مقایسه شاخص­های سلامت یک مکان در زمان­های مختلف اختصاص دارد که سعی در حذف مخدوش­کننده­های فردی دارد، گرچه انواع دیگری از مخدوش­کننده­ها هم­چنان مشکل آفرین خواهند بود. هم­چنین این دسته از مطالعات قادر به بررسی اثرات مواجهه طولانی مدت با آلاینده­ها نیستند. مشکلات دیگری در زمینه این قبیل مطالعات وجود دارد که تا حدودی همه انواع مطالعات را تحت تأثیر خود قرار می­دهند: نواقص موجود در اندازه­گیری مناسب مواجهه، همزمانی مواجهه با چند آلاینده و اثرات متقابل مواجهه با آن­ها، تحرک افراد بین محیط­های مختلف با غلظت­های مختلف (به خصوص بین فضاهای بسته و باز) و مشکل چگونگی تخصیص مواجهه به آن­ها، مشکلات موجود در اندازه­گیری سطوح الاینده­های درون فضاهای بسته و... نتیجه­گیری: علی­رغم پیشرفت قابل ملاحظه در یافته­ها و روش­شناسی مطالعات اپیدمیولوژی، هنوز هم مشکلات اساسی در بررسی اثرات سلامت حاصل از مواجهه با آلودگی هوا وجود دارد.

Title: تأثیرعوامل محیطی و فردی بر بار اقتصادی مستقیم تحمیل شده بر بیماران دیابتی نوع دو در ایران: کاربردی از مدل رگرسیون چندسطحی تعمیم یافته

http://knh.shmu.ac.ir/index.php/site/article/view/967

مقدمه: دیابت شیرین پرهزینه­ترین بیماری دنیا شناخته می­شود. بخشی ازهزینه­های این بیماری مانند درصدی از هزینه­های خرید دارو و خدمات درمانی بیمارستانی توسط یارانه دولتی پرداخت می­شود، اما بخش اعظم آن توسط خود بیمار و از درآمد وی هزینه می­گردد. از طرفی پیش­بینی می­شود هر چقدر محل سکونت بیمار از نظر امکانات و خدمات محروم­تر باشد، میزان کسر و جذب درآمد ماهیانه وی به­دلیل مشکلات مربوط به بیماری بیش­تر خواهد بود و در نتیجه بار اقتصادی بیش­تری برب یمار تحمیل خواهد شد. لذا هدف از این مطالعه تعیین ارتباط عوامل محیطی و فردی بر بار اقتصادی تحمیل شده بر بیماران دیابتی نوع دو در ایران است. مواد و روش­ها: داده­های این گزارش از مطالعه مقطعی که در سال 1385 در کل کشور بر روی 4000 بیمار دیابتی به روش مصاحبه انجام شد، استخراج شده است. متغیر وابسته یا بار مستقیم دیابت جذب بیش از 30% درآمد ماهیانه به­دلیل دیابت تعریف گردید. در سطح فردی (سطح یک) متغیرهای وضعیت سلامتی، وضعیت اقتصادی، وضعیت بیمه، تعداد سال­های آگاهی از بیماری، نوع واحد ارايه­دهنده خدمات درمانی و متغیرهای زمینه­ای شامل جنس، سن، وضعیت تحصیل، شغل و تأهل و در سطح محیطی (سطح دو- استان) تعداد مراکز درمانی، تعداد پزشک متخصص، نسبت باسوادی، نرخ بیکاری و درصد شهرنشینی مورد مطالعه قرارگرفتند. به­منظور تعیین تأثیر عوامل فردی و محیطی بر بار اقتصادی تحمیل شده بر بیماران از مدل رگرسیون لجستیک دو سطحی و نرم­افزار  MlWin2.1استفاده شد. یافته­ها: از 4000 فرد مورد مطالعه 3234 نفر پرسش­نامه را به­طورکامل پرکردند (درصد پاسخگویی 80%). از این تعداد 3/61% زن و 7/38% مرد با میانگین سنی کل 4/59 سال (انحراف معیار 8/11) بودند. یافته­ها نشان داد از بین متغیرهای سطح دو متغیرنسبت باسوادی در استان OR=0.9,95%CI:0.83-0.98)) و از بین متغیرهای سطح یک متغیرهای وضعیت سلامتی OR=0.23,95%CI:0.18-0.30))، وضعیت اقتصادی، جنس، تعداد سال­های آگاهی از بیماری، نوع منطقه سکونت OR=1.33,95%CI:1.02-1.72)) و نوع واحد دریافت درمان ارتباط معنادار با بار اقتصادی داشتند. از سایر متغیرهای سطح دو درصد شهرنشینی و وجود تعداد پزشک متخصص ارتباط معکوس با بار مستقیم بیماری داشتند اما معنادار نبودند. نتیجه­گیری: علاوه بر عومل فردی، باید عوامل محیطی نیز در مدل­سازی هزینه­های دیابت در نظر گرفته شوند. در این مطالعه، فرضیه مبنی بر ارتباط محرومیت محل سکونت (درصد بی­سوادی) بیمار با هزینه­های تحمیلی دیابت بر بیمار تأیید گردید. توجه به عوامل محیطی در سیاست­های بهداشتی می­تواند کاهش تحمیل هزینه­های مستقیم دیابت بر بیماران را به­دنبال داشته باشد.

Title: محاسبه هزينه تمام شده خدمات ارايه شده در مراكز بهداشتي درماني و خانه¬هاي بهداشت شهرستان شاهرود در سال 1387

http://knh.shmu.ac.ir/index.php/site/article/view/971

مقدمه: محاسبه هزينه­ها ابزار مديريتي مهمي جهت برنامه­ريزي، كنترل، نظارت و ارزشيابي خدمات سلامت است كه به­منظور تصميم­گيري آگاهانه انجام مي­شود. مطالعه به­منظور تعیین هزينه تمام­شده خدمات ارايه شده در مراكز بهداشتي درماني و خانه­هاي بهداشت شهرستان شاهرود در سال 1387 انجام شد. مواد و روش­ها: اين مطالعه از نوع پژوهش­هاي کاربردی است که به شیوه توصيفي- مقطعي انجام شد. در اين مطالعه کلیه مراکز بهداشتی درمانی شهری و روستایی به روش سرشماری مورد پژوهش قرارگرفت. اطلاعات مورد نياز در قالب فرم­هاي جمع­آوري داده­ها گردآوري و وارد نرم­افزار تحلیل هزینه شد. جهت تحليل داده­ها از نرم­افزار تحلیل هزینه، Excel و SPSS استفاده گردید. يافته­ها: یافته­های حاصل از تحلیل داده­ها نشان داد که 43/44 درصد ارايه­دهندگان را مردان و 57/55 درصد را زنان تشکیل می­دادند. 82/22 درصد از نیروها در خانه­های بهداشت، 07/26 درصد در مراکز بهداشتی درمانی روستایی، 09/9 درصد در مراکز بهداشتی درمانی شهری، 53/24 درصد در مراکز بهداشتی درمانی شبانه­روزی شهری، 57/2 درصد در پایگاه­های بهداشتی و 92/14 درصد در ستاد معاونت بهداشتی شهرستان شاغل بودند. هزینه­های پرسنلی با 13/66% و پس از آن هزینه ستادی شهرستان با 85/12%، دارو با 95/10% و مصرفی اختصاصی با 76/3% قرار داشتند. بالاترین هزینه خدمت مربوط به آموزش بهورز با 1325209 ريال و کم­ترین آن مربوط به نمونه­گیری آنفلوانزا با 3872 ریال بود. نتيجه­گيري: با توجه به بالابودن هزینه­هاي پرسنلي، افزايش بهره­وري نيروي كار نقش مهمي در كاهش مخارج خواهد داشت. هم­چنين تعدیل نیروها و استفاده از مشارکت بخش خصوصی در ارائه خدمات و برون­سپاری واحدهاي پرهزينه می­تواند در استفاده بهینه از منابع نقش مهمی را ایفا نماید.

Title: برآورد بار قابل انتساب به عوامل خطر سکته مغزی در ایران

http://knh.shmu.ac.ir/index.php/site/article/view/972

مقدمه: اطلاع از مقدار بار قابل انتساب به عوامل خطر سکته مغزی به­دلیل نقش آن در تدوین راه­کارهای پیشگیری و اولویت­بندی مداخلات ضروری است. مطالعه حاضر با هدف برآورد بار قابل انتساب به برخی از عوامل خطر سکته مغزی شامل فشارخون بالا، اضافه وزن، چاقی، مصرف سیگار و فعالیت بدنی ناکافی در ایران انجام شده است. مواد و روش­ها: به­منظور محاسبه سهم تأثیر بالقوه Potential Impact Fraction)) و درصد بار قابل انتساب به عوامل خطر سکته مغزی از متدولوژی ارزیابی خطر مقایسه­ای (Comparative Risk Assessment) سازمان جهانی بهداشت استفاده گردید. اطلاعات مربوط به شیوع عوامل خطر از پنجمین دوره بررسی کشوری عوامل خطر بیماری­های غیرواگیر در سال 1388 استخراج گردید. سهم تأثیر بالقوه در دو حالت حداقل سطح خطر تئوریک و امکان­پذیر محاسبه گردید و از رویکرد شبیه­سازی (Simulation) برای برآورد عدم قطعیت (Uncertainty) درصد بار قابل انتساب استفاده گردید. يافته­ها: با کاهش شیوع فشارخون بالا از 16 درصد در مردان و 1/16 درصد در زنان به 10 درصد، به ترتیب7/15 درصد و 8/15 درصد از کل سال­های از دست رفته تعدیل شده با ناتوانی (DALYs) بدلیل سکته مغزی در مردان و زنان را می­توان کاهش داد. به­طور کلی با حذف مواجهه مردان بالای 15 سال کشور با عوامل خطر فشارخون بالا، اضافه وزن، چاقی، مصرف سیگار و فعالیت بدنی ناکافی به­ترتیب 6/41، 12، 8/4، 4/3 و 8/2 درصد از کل سال­های از دست رفته تعدیل شده با ناتوانی به­دلیل سکته مغزی در مردان قابل اجتناب خواهد بود. این مقدار برای زنان به ترتیب 7/41، 9/10، 9/10، 2/0 و 8/4 درصد است. نتیجه­گیری: اگرچه در این مطالعه شیوع عوامل خطرساز سکته مغزی از گزارش پنجمین دوره نظام مراقبت عوامل خطر بیماری­های غیرواگیر در سال 1388 گرفته شده است، با این وجود به­منظور تصمیم­گیری بهتر و متقاعد نمودن سیاستگزاران نظام سلامت پیشنهاد می­گردد گزارش بار قابل اجتناب (Avoidable Burden) براساس مطالعات بروز شده محاسبه بار بیماری در سطح ملی و به­صورت تعداد سال­های از دست رفته تعدیل شده با ناتوانی بجای درصد بار قابل انتساب انجام گیرد.

Title: تخمین موارد ایدز در کشور با استفاده از مدل¬سازی، 1388 تا 1392

http://knh.shmu.ac.ir/index.php/site/article/view/973

مقدمه: به­منظور ارزیابی اثر مداخلات و ایجاد برنامه کنترل منطقی در زمینه HIV، تخمین تعداد موارد مثبت HIV از اهمیت بالایی برخوردار است. مواد و روش­ها: با استفاده از مرور ساختارمند، تمامی مقالات منتشر شده و متون منتشر نشده به زبان فارسی و انگلیسی مورد بررسی قرار گرفتند. سپس پارامترهای مورد نیاز برای مدل­سازی استخراج شد. استخراج داده­ها و ارزیابی معیارهای ورود به مطالعه به­طور مستقیم تحت نظارت گروه کارشناسان از مرکز کنترل بیماری­های واگیر در ایران و مرکز آموزش نظام مراقب HIV/ ایدز انجام شد. با استفاده از نرم­افزارهای EPP و Spectrum، به تخمین موارد مثبت HIV در ایران تا سال 1392 پرداخته شد. به­دلیل عدم قطعیت بعضی از پارامترهای اساسی در سطح ملی، چهار سناریو متفاوت ایجاد گردید. يافته­ها: باوجود اینکه خروجی چهار سناریوی ایجاد شده به­طور قابل ملاحظه­ای تفاوت داشت، الگوی تغییرات کمابیش قابل قیاس بود و نشان داد که در گذشته تزریق دارو راه اصلی انتقال بوده است. ولی به­هر حال، در طول سال­های اخیر و در آینده، اغلب موارد انتقال در بین زنان تن­فروش و مشتریان آن­ها خواهد بود و اغلب موارد از جمعیت عادی خواهند بود (حداقل 62% در سال 1388). براساس تخمین­های آخرین سناریو، تعداد افراد مثبت از لحاظ HIV 88 هزار نفر در سال 1388 بوده و به 105هزار نفر در سال 1392 افزایش می­یابد. در سال 1388، 6570 مورد جدید عفونت (فاصله اطمینان 95%: 52700-1080) دیده شد و 5800 مورد مثبت HIV (فاصله اطمینان 95%: 30700-4040) فوت کردند. نتیجه­گیری: این مطالعه نشان داد که انتقال HIV در بین معتادان تزریقی در سال­های اخیر کاهش پیدا کرده است که می­تواند به­دلیل مداخلات اخیر باشد. به­هر حال، در این مطالعه نشان دادیم که تعداد موارد مثبت HIV در سال­های آتی افزایش خواهد یافت. پیشنهاد می­شود که به­منظور کاهش انتقال هتروسکسؤال در آینده نزدیک، مداخلات مخصوص با هدف جمعیت عادی و زیرگروه­های در معرض خطرانجام شود.

Title: ارزیابی سریع نظام ثبت وقایع حیاتی و نظام آمار حیاتی (تولد و مرگ) ایران بر اساس مدل پیشنهادی سازمان جهانی بهداشت

http://knh.shmu.ac.ir/index.php/site/article/view/974

مقدمه: داده­های ثبت شده وقایع حیاتی پايه نظام آمارهاي وقايع حياتي را شكل مي­دهند كه قسمت مهم نظام اطلاعات سلامت كشورها را شامل می­شوند. این داده­ها می­توانند برای شناسایی اولویت­ها، سنجش نابرابری سلامت و ارزشیابی برنامه­های سلامت به­کار روند. در کشورهای پیشرفته وقايع ثبتي) ثبت احوال(، بهترين منبع داده­هاي آمار حياتي هستند، ولي اين نظام معمولاً در بسياري از كشورهاي جهان سوم ضعيف و ناكامل هستند. هدف این مطالعه ارزیابی سریع نظام ثبت وقایع حیاتی و نظام اطلاعات حاصل از آن در خصوص تولد و مرگ ایران بر اساس مدل پیشنهادی سازمان جهانی بهداشت می­باشد. مواد و روش­ها: ابزار مورد استفاده در این ارزیابی سریع، شامل 25 سوال در خصوص عملکرد نظام وقایع ثبت و آمار حیاتی می­باشد. سؤالات در 11 گروه طبقه­بندی شده­اند. برای پاسخ به هر سؤال کشورها می­توانند از بین 4 سناریو طراحی شده برای هر سؤال، یکی را که با شرایط نظام ثبت وقایع حیاتی آن کشور همخوانی دارد، انتخاب کنند. پاسخ هر سؤال، نمره­ای بین صفر تا 3 دارد. با توجه به مدل پیشنهادی، برای ارزیابی مورد نظر جلسه­ای متشکل از مسوولین و کارشاسان بخش­های مختلف از جمله مرکز آمار ایران، وزارت بهداشت و آموزش پزشکی، سازمان ثبت احوال کشور و پزشکی قانونی تشکیل شد و ارزیابی مورد نظر بر اساس مدل پیشنهادی انجام گرفت. یافته­ها: نتایج این ارزیابی سریع نشان داد نمره نهایی برای وضعیت نظام ثبت وقایع حیاتی و نظام آمار حاصل از آن در کشورمان، 53 از 100 می­باشد که براساس مدل ارایه شده که نتایج را در چهار گروه طبقه­بندی کرده است، در گروه ضعیف قرار می­گیرد. نتایج هم­چنین نشان داد که بسیاری از ابعاد ثبت وقایع حیاتی و نظام آمار حاصل از آن در کشورمان به نحوه مطلوبی عمل نمی­کنند و نیاز به مداخلات چندگانه دارند. نتیجه­گیری: با توجه به اهمیت آمار وقایع حیاتی در نظام اطلاعات سلامت، براساس مدل ارایه شده برنامه­ریزی جامع که می­بایست به دنبال این ارزیابی سریع انجام شود از اقدامات اساسی برای ارتقای نظام اطلاعات وقایع حیاتی کشورمان می­باشد.

Title: مطالعه تک نمونه¬ای (single subject design) با استفاده از روش آماره C (C statistic)

http://knh.shmu.ac.ir/index.php/site/article/view/975

مقدمه: در مواردی­که در یک مطالعه مداخله­ای گرفتن گروه کنترل غیراخلاقی بوده و یا تعداد نمونه بسیار کم است (بیماری­های نادر)، لازم است حداقل در مراحل اولیه، مطالعه­ای تک نمونه­ای انجام گیرد. جهت آنالیز این مطالعات از دو روش کلی گرافیکی مشاهده­ای و استنتاجی آماری استفاده می­شود که در این بین روش آماره C یک روش بسیار پیشرفته و با توان بالا محسوب می­شود. مواد و روش­ها: ابتدا برای روند داده­ها در فاز پایه آماره C محاسبه و در مورد معناداری آماری روند مذکور تحلیل صورت می­گیرد. اگر روند از نظر آماری معنادار نباشد آنوقت داده­های مربوط به فاز پایه و مداخله با هم تجمیع شده و مجدداً آماره C محاسبه و معناداری آماری آن مورد بررسی قرار گرفته و در صورت معنادار بودن دلیل بر اثربخشی مداخله مورد مطالعه می­باشد. هم­چنین با ذکر یک مثال واقعی (با شرایط عدم معناداری روند در فاز پایه) که بر اساس قسمتی از داده­های مطالعه بررسی تأثیر استفاده از روش آی- تی- ام- تی (ITMT) بر روی مهارت­های درون دستی دو کودک همی پلژی خفیف انجام شده است و هم­چنین یک مثال فرضی (با شرایط معناداری روند در فاز پایه) روش مذکور مورد بحث قرار می­گیرد. یافته­ها: در فاز پایه روند تغييرات كليه پارامترهاي كيفيت و عملكرد مهارت­هاي دستي (8 پارامتر) در مثال اول معنادار نبوده ولي روند تغييرات تركيبي از اندازه­هاي فاز پايه و فاز مداخله معنادار بوده است. (P<0.05; Z score> 1.68) و هم­چنین در مثال دوم هم در فاز پایه روند تغييرات كليه پارامترهاي كيفيت و عملكرد مهارت­هاي دستي (8 پارامتر) معنادار بوده و هم روند تغييرات تركيبي از اندازه­هاي فاز پايه و فاز مداخله معنادار بوده است (P<0.01; Z score>2.58). نتیجه­گیری: مطالعه تک نمونه­ای و آنالیز آن با استفاده از روش آماره C، علي­رغم محدوديت­هاي خاص خود، امکان ارزیابی اثر مداخلات را در شرایط خاص، فراهم می­آورد.

Title: امید به زندگی روستانشینان ایران براساس داده¬های تصحیح شده زیج حیاتی سال 1387

http://knh.shmu.ac.ir/index.php/site/article/view/976

مقدمه: امید به زندگی یکی از مهم­ترین اندازه­های خلاصه­ای(Summary Measures) است که به­منظور مقایسه میزان­های مرگ در یک کشور، بین کشورها و هم­چنین در زمان­های مختلف مورد استفاده قرار می­گیرد. این مطالعه با هدف برآورد امید زندگی و شاخص­های میرایی در جمعیت روستایی کشور با استفاده از زیج حیاتی طراحی شده است. بدین منظور با استفاده از جدیدترین روش­های محاسبه امید به زندگی که توسط سازمان بهداشت جهانی ارايه شده برای اولین بار در کشور به تصحیح داد­های زیج حیاتی پرداخته و امید به زندگی بدو تولد محاسبه شده است. مواد و روش­ها: لازمه محاسبه امید به زندگی با استفاده از جداول عمر دوره­ای میزان­های میرایی اختصاصی سنی است. برای برآورد صحیح میزان­های مرگ­ومیر لازم است که داده­ها از نظر کم شماری تصحیح گردند. به­منظور اصلاح کم شماری میزان مرگ­ومیر بزرگسالان از روش تعادل رشد براس(Growth Balance Method) استفاده شده است که این روش برای گروه سنی زیر 5 سال مناسب نیست، به همین دلیل با استفاده از نتایج مطالعه سلامت و جمعیت (DHS) سال 1379 به تصحیح کم شماری میزان مرگ برای این گروه سنی پرداخته شده است. يافته­ها: در این مطالعه میزان مرگ تصحیح شده کودکان زیر 5 سال در پسران و دختران به ترتیب 4/29 و 1/21 در هزار تولد زنده بوده است. خطر مرگ تصحیح شده بزرگسالان 15 تا 59 ساله در مردان و زنان به­ترتیب 162/0 و 098/0 بوده است. امید به زندگی بدو تولد تصحیح شده در مردان 72/71 و در زنان 6/74 سال بوده است.از نظر درصد کامل بودن داده­های مرگ زیج حیاتی مقدار این شاخص برای مردان90% و برای زنان 7/78% بوده است. نتیجه­گیری: با توجه به نتایج مطالعه حاضر می­توان گفت که داده­های مرگ گزارش شده زیج حیاتی منبع مناسبی برای محاسبه امید به زندگی و سایر شاخص­های مرگ­ومیر می­باشد و می­توان از آن برای مقایسه میزان­های مرگ بین استان­های مختلف و طی سالیان گذشته استفاده نمود.

Title: کاربرد تحقیقات کیفی و ترکیبی به¬عنوان رویکردهای نوین در اپیدمیولوژی اجتماعی

http://knh.shmu.ac.ir/index.php/site/article/view/977

مقدمه: رشته اپیدمیولوژی به­عنوان علم میان رشته­ای دارای شاخه­های متعدد از جمله اپیدمیولوژی اجتماعی است.این شاخه­ی اپیدمیولوژی ترکیبی از اپیدمیولوژی کلاسیک و علوم اجتماعی و رفتاری است. نگرش اپیدمیولوژی اجتماعی به فاکتورهای اجتماعی صرفاً از زاویه جسمانی نیست بلکه نگرشی مبتنی بر هویت مستقل عوامل اجتماعی و مطالعه نقش آن­ها بر سلامت جامعه می­باشد.از این روی، به نظر می­رسد که اپیدمیولوژی اجتماعی پتانسیل استفاده از رویکردهای متعدد کمی، کیفی و ترکیبی را داراست. هدف این مطالعه ارزیابی کاربرد این رویکردها در تحقیقات اپیدمیولوژی اجتماعی می­باشد. مواد و روش­ها: در این مطالعه از روش مرور غیرساختاریافته استفاده و با به­کارگیری کلمات کلیدی "رویکردهای تحقیقات کیفی، روش ترکیبی، سلامت، فاکتورهای اجتماعی و اپیدمیولوژی اجتماعی" به زبان انگلیسی از منابع متعدد الکترونیکی و کتابخانه­ای در دو دهه گذشته مورد جستجو قرار گرفت. منابع قابل دسترس شامل کتاب­ها، مقالات، گزارش­های سمینارهای علمی در حوزه­های مشترک کلید واژه­ها مورد بررسی قرار گرفت. يافته­ها: بررسی تحقیقات و تألیفات قبلی نشان­دهنده استقبال محققین از این رویکردها (کیفی و ترکیبی) در حوزه­های مختلف علوم پزشکی در درک معضلات سلامت می­باشد. مضافاً اپیدمیولوژیست­های اجتماعی معتقدند که مطالعات کمی وکیفی باید به موزات هم نگریسته شوند زیرا بدون خلق تئوری­ها و مدل­ها، اطلاعات پایه اندکی در رابطه با پارامترهای مورد اندازه­گیری، کاربرد مدل­های آماری و چگونگی تفسیر نتایج آن­ها موجود می­باشد. نتيجه­گيري: به نظر می­رسد که اپیدمیولوژی به­طور اعم و اپیدمیولوژی اجتماعی به­طور اخص نیازمند به تحول در به­کارگیری رویکردهای مذکوردر حوزه­های مختلفی چون روانی، اجتماعی و معنوی بوده، تا با کاربرد این رویکردها علاوه بر انعطاف­پذیری محققین سلامت و علوم پزشکی در استفاده از رویکردهای متعدد، باعث افزایش توانایی آنان در درک مشکلات سلامت از جمله بیماری­ها و معضلات بهداشتی نوپدید گردد.

Title: سرمایه اجتماعی و عوامل تعیین¬کننده آن در شهر تهران

http://knh.shmu.ac.ir/index.php/site/article/view/978

مقدمه: سرمایه اجتماعی از مفاهیم نوینی است که امروزه در بررسی­های اقتصادی و اجتماعی جوامع مدرن مطرح گردیده است. می­توان ادعا کرد سرمایه اجتماعی نقشی مهم­تر از سرمایه فیزیکی و انسانی دارد چرا که در غیاب آن سایر سرمایه­ها کارا نخواهد بود، بر خلاف کشورهای توسعه یافته، در کشورهای در حال توسعه از جمله ایران، سرمایه انسانی به­ویژه سرمایه اجتماعی مبهم باقی مانده است. با توجه به نقش کلیدی این سرمایه درتمام زمینه­ها و نگرش­های مختلف نسبت به آن در تمام جنبه­های زندگی که افزایش پژوهش­های مرتبط با این مهم حاکی از این جایگاه می­باشد و علاوه بر آن با توجه به تناقض در مطالعات قبلی، در این تحقیق ما بر آنیم که در ابتدا به بررسی و سنجش سرمایه اجتماعی در اولین کلانشهر یعنی شهر تهران پرداخته و با بهره­گیری از مدل­های نظری به تعبیر این مهم بپردازیم عمده­ترین عوامل مؤثر بر آن کدامند تا بتوان از نتایج حاصل از این بررسی به­منظور سنجش سرمایه اجتماعی و روند تغییرات آن در ایران بهره برد. مواد و روش­ها: روش تحقیق از نوع پیمایشی و از نظر زمانی در یک مقطع زمانی انجام می­گیرد. واحد تحقیق فرد از خانوار می­باشد. جمعیت مورد مطالعه، جمعیت ساکن در مناطق 22 گانه شهر تهران می­باشند که اطلاعات دموگرافیک و شغل و تحصیلات و درآمد و اطلاعات سرمایه اجتماعی شامل مشارکت­های داوطلبانه، فعالیت دسته جمعی، اعتماد، احساس تعلق و پای­بندی به ارزش­ها در 22300 نفر توسط 2 پرسش­نامه جداگانه طی طرح بررسی سنجش نابرابري­ها در شهر تهران URBAN-HEART)) جمع­آوری شده است. يافته­ها: سطوح مختلف سرمایه اجتماعی در مناطق مختلف شهر تهران مقدارهای مختلفی را به خود اختصاص داد تمام مؤلفه­های اندازه­گیری شده سرمایه اجتماعی در دو جنس اختلاف معناداری را از لحاظ آماری نشان داد، هم­چنین غیر از فعالیت دسته­جمعی، سایر شاخص­های سرمایه اجتماعی در گروه­های مختلف سنی تفاوت مثبت معنادار آماری را نشان داد. همه متغیرهای سرمایه اجتماعی با هزینه­های مصرفی خانوار ارتباط مثبت معناداری را نشان داد. در این مطالعه تمام مؤلفه­های سرمایه اجتماعی به جز مشارکت در فعالیت­های داوطلبانه با وضعیت تأهل ساکنان شهر اختلاف معناداری را نشان داد. غیر از فعالیت دسته جمعی، سایر شاخص­های سرمایه اجتماعی در گروه­های مختلف  بر حسب تحصیلات در تفاوت مثبت معنادار آماری را نشان داد. وضعیت اشتغال، مالکیت منزل مسکونی، بعد خانوار، مدت اقامت در منزل مسکونی از سایر عوامل مؤثر بر برخی مؤلفه­های سرمایه اجتماعی بودند. نتیجه­گیری: عوامل مختلف دموگرافیک، تأثیرات مختلفی را به­صورت مثبت یا منفی در مؤلفه­های مختلف سرمایه اجتماعی از خود بروز دادند. در نهایت نمی­توان از اثر تجمعی عوامل چندگانه بر عناصر مفهومی سرمایه اجتماعی غافل شد، پس انتظار می­رود به­دنبال بهبود عوامل مؤثر قابل تعدیل، نظیر وضعیت اقتصادی، تحصیلات، نقش سایر عوامل نظیر نسل و جنسیت را در جهت اثرگذاری مثبت روی این سرمایه عظیم انسانی را تحت­الشعاع قرار داد.

Title: محاسبه بار منتسب به اضافه وزن و چاقی در بیماری¬های غیر واگیر در استان مرکزی سال 1385

http://knh.shmu.ac.ir/index.php/site/article/view/979

مقدمه: سازمان بهداشت جهانی به­طور مداوم كشورها را به محاسبه­ی بار بيماري­ها در سطح ملی به­عنوان بهترين راهنما جهت سياست­گذاری درست در سيستم سلامت تشویق می­کند. لذا مطالعه­ای به­منظور تعیین مقدار بار منتسب به عوامل خطر بیماری­های غیر واگیر به اجرا درآمد. مواد و روش­ها: داده­های مرگ ثبت شده در استان مرکزی، بر اساس روش ارزیابی خطر مقایسه­ای سازمان جهانی بهداشت جهت محاسبه YLL استفاده شد و پس از محاسبه خطر قابل انتساب به اضافه وزن و چاقی، بار منتسب به هر بیماری جداگانه محاسبه گردید. يافته­ها: از کل 7176 مورد مرگ ثبت شده، 252214 سال عمر به­دلیل مرگ زودرس از دست رفته بود. مجموع سال­های از دست رفته عمر به­دلیل مرگ زودرس در زنان 102245 سال و در مردان 149969 سال به­دست آمد. سهم علل اصلی سال­های از دست رفته عمر به­دلیل مرگ زودرس استان به­ترتیب در بیماری­های قلبی عروقی 89/40%، حوادث 61/17%، سرطان­ها 46/10% محاسبه شد. نتیجه­گیری: این مطالعه اهمیت و ضرورت تشخیص اضافه وزن و چاقی را به­عنوان یکی از عوامل خطر عمده برای بیماری­های غیر واگیر به­ویژه در زنان نشان داد که نیاز به توسعه و به کار بردن و ارزیابی مداخله­های جامع برای کاهش اثر اضافه وزن و چاقی یک ضرورت است

Title: بررسي رابطه شاخص¬هاي عدالت اجتماعي و سلامت در ايران؛ 1385-1363

http://knh.shmu.ac.ir/index.php/site/article/view/980

مقدمه: دستيابي به عدالت اجتماعي و ارتقاء سلامت جامعه از جمله مهم­ترين اهداف و محورهاي سياست­هاي توسعه­اي (توسعه اجتماعي) در جوامع مي­باشد. بر اين اساس بررسي شاخص­هاي اين دو محور اصلي توسعه اجتماعي حائز اهميت مي­باشد. بررسي رابطه شاخص­هاي عدالت اجتماعي و سلامت در ايران در سال­هاي 1385-1363. مواد و روش­ها: مطالعه تحليلي و از نوع همبستگي اكولوژيكي بوده و واحد تحليل جمعيت كشور در سال­هاي مورد بررسي مي­باشد. داده­هاي مورد نياز مطالعه از مركز آمار ايران و بانك مركزي تهيه شده و توسط نرم­افزارهاي STATA و SPSS پردازش و تحليل شدند. يافته­ها: در سطح معناداري (01/0P<) رابطه شاخص­هاي عدالت اجتماعي با اميد به زندگي معنادار بود (0001/0P=): سهم درآمدي (69/0-R=)، فقر (71/0-R=)، هزينه در بهداشت (79/.R=)، هزينه در آموزش (85/0R=). در سطح معناداري (01/0P<) رابطه شاخص­هاي عدالت اجتماعي با مرگ­ومير كودكان زير 5 سال معنادار بود (0001/0P=): سهم درآمدي (71/.R=)، فقر (64/0R=) هزينه در بهداشت (69/0-R=)، هزينه در آموزش (76/0- R= ). نتيجه­گيري: افزايش فقر و نابرابري درآمدي اثر معكوس بر اميد به زندگي داشته و با ميزان مرگ­ومير كودكان زير 5 سال رابطه مستقيم دارد. افزايش هزينه­هاي دولت در آموزش و بهداشت با اميد به زندگي رابطه مستقيم و با مرگ­ومير كودكان زير 5 سال رابطه عكس دارد. براساس آزمون رگرسيون، نابرابري درآمدي و هزينه در آموزش به تنهاي (52F=)، 86 درصد تغيرات متغير سلامت را تبين كردند.

Title: رده¬بندی وضعیت بهداشت و سلامت مادران روستایی در استان¬های مختلف ایران با استفاده از روش¬های چند متغیره عاملی و خوشه¬ای

http://knh.shmu.ac.ir/index.php/site/article/view/981

مقدمه: تأمین سلامت مادران به­عنوان یکی از گروه­های آسیب­پذیر از اهمیت زیادی در حوزه مراقبت­های بهداشتی و درمان برخوردار است. هدف از مطالعه حاضر رتبه­بندی و شناسایی استان­های همگن به لحاظ شاخص­های بهداشتی و جمعیتی سلامت مادران است. شناسایی جایگاه استان­ها نسبت به یکدیگر در برنامه­ریزی­های منطقه­ای و تخصیص منابع براساس الویت­ها و کاهش نابرابری­ها راهبردی عملی و اجرایی خواهد داشت. مواد و روش­ها: در این پژوهش استان­های کشور به لحاظ برخی شاخص­های بهداشتی و جمعیتی مرتبط با سلامت مادران روستايي بر گرفته از زیج حیاتی سال 85 با استفاده از تحلیل عاملی و تحلیل خوشه­ای رتبه­بندی و دسته­بندی شده­اند. تجزیه و تحلیل­ها در نرم­افزارهای SPSS و Minitab صورت گرفته است. یافته­ها: با استفاده از تحلیل عاملی با تلخیص شاخص­های متنوع عاملی که معرف مناسبي از سطح سلامت مادران در استان­های مختلف بود به­دست آمد و استان­های کشور براساس آن رتبه­بندی گردید. بر اساس این رتبه­بندی استان­های گیلان، مازندران و تهران از وضعیت مناسبی در حوزه سلامت مادران برخوردارند اما استان­های خوزستان، کهکیلویه و بویراحمد، و هرمزگان از وضعیت مناسب بی­بهره­اند، در ضمن استان سیستان و بلوچستان در وضعیتی وخیمی قرار دارد. در تکمیل نتایج به­دست آمده از تحلیل عاملی با استفاده از تحلیل خوشه­ای استان­های همگن نيز شناسایی شده است. نتیجه­گیری: روش­های تحلیل چند متغیره ابزاری مؤثر در رده­بندی و شناسایی استان­های همگن به لحاظ شاخص­های سلامت مادران است. نتایج حاصل از این تحقیق در برنامه­ریزی در خصوص نحوه اجرا و تخصیص خدمات بهداشتی به مادران روستایی، برای دست­اندرکاران امور بهداشتی و درمانی کشور مفید خواهد بود.

Title: شیوع کمبود ید در زنان باردار تحت پوشش مراکز بهداشتی- درمانی شهرستان ارومیه

http://knh.shmu.ac.ir/index.php/site/article/view/982

مقدمه: تقریباً 50% مردم جهان کم­تر از حد مورد نیاز ید دریافت می­کنند. این نسبت بزرگ از جمعیت جهان در نواحی گسترده­ای که غالباً کوهستانی نیز هستند زندگی می­کنند. نوع و شدت عوارض کمبود ید در گروه­های مختلف جمعیتی متفاوت است ولی حساسیت و آسیب­پذیری زنان باردار و شیرده و هم­چنین جنین یا نوزاد آن­ها به علت وابستگی در تأمین ید و هورمون­های تیروئیدی به مادر حائز اهمیت ویژه­ای است. مواد و روش­ها: به منظور بررسی سطح ید دریافتی زنان باردار شهرستان ارومیه، 490 زن باردار که کم­تر از 12 هفته از شروع بارداری آن­ها گذشته بود از 12 منطقه شهری و روستایی وارد مطالعه شدند. اطلاعات جمعیت شناختی با تکمیل پرسش­نامه به­دست آمد. نمونه­های ادرار در دمای (20-) درجه سانتیگراد تا زمان تکمیل حجم نمونه نگهداری و سپس میزان ید ادرار در نمونه­های به­دست آمده سنجش شد. نمونه نمک خوراکی خانوارها نیز تهیه و ید سنجی شدند. يافته­ها: میانه ید ادرار به­طور کلی در این شهرستان 73.5 میکروگرم در لیتر بود. بین برخی نواحی شهری و روستایی تفاوت معنادار در متوسط ید ادرار دیده شد. شیوع کمبود ید متوسط و شدید 3/33% بود. ارتباط معناداری بین گروه­های سنی و مبدأ جغرافیایی با میزان ید ادرار دیده نشد. 95.7% نمونه­های نمک، ید کافی (حداقل 15 ppm) داشتند. نتيجه­گيري: با توجه به شرایط ویژه بارداری، تنها مصرف نمک یددار نمی­تواند تأمین کننده ید مورد نیاز زنان باردار باشد و ارايه مکمل ید در مناطقی که کمبود ید وجود دارد، با توجه به شدت این کمبود، برای گروه­های خاصی ضروری است.

Title: بررسی ارتباط بین وزن هنگام تولد و وضعیت تغذیه و ابتلا به بیماری قبل از یک سالگی با وضعیت رشد كودكان 5-2 ساله شهر زاهدان سال87-1386

http://knh.shmu.ac.ir/index.php/site/article/view/983

مقدمه: ارزیابی دوره‌اي شاخص­هاي تغذیه­ای در کودکان و تعیین عوامل مرتبط با آن در ارتقاء سلامت کودکان و جامعه اهمیت بسزایی دارد و وضعیت بهداشتی هر منطقه را نشان می­دهد. هدف از اين مطالعه بررسی ارتباط بین وزن هنگام تولد و وضعیت تغذیه و ابتلا به بیماری قبل از یک سالگی با وضعیت رشد كودكان 5- 2 ساله شهر زاهدان می­باشد مواد و روش­ها: در اين مطالعه مقطعي تعداد 1012 كودك 5- 2 سال از 5 منطقه شهري به­صورت چند مرحله­اي انتخاب و بررسي شدند. اطلاعات با استفاده از پرونده­ها و مصاحبه با مادر كودك مراجعه­كننده جمع­آوري شد. در اين مطالعه سه شاخص وزن براي قد، قد براي سن و وزن براي سن به ترتيب به­عنوان نمايه وضع تغذيه زمان حال يا لاغري، زمان گذشته يا كوتاه قدي و زمان حال و گذشته يا كم وزني با Z کم­تر از 5/2-مورد بررسي قرار گرفت. داده­ها در نرم­افزار SPSS با استفاده از رگرسیون لوجستیک تک متغیره مورد تجزیه و تحلیل قرار گرفت. يافته­ها: در این مطالعه میزان شیوع لاغری، کوتاه قدی وکم وزنی به­ترتیب 9/2%، 4/8% و 4/2% برآورد شد. در اين مطالعه 6/28% کودکان سابقه عفونت داشتند که از بين آن­ها 7% به اسهال،2/10% به عفونت­هاي تنفسي و 5/5% نیز به ساير بيماري­ها مبتلا شده بودند. در ضمن 4/11% از كودكان به­طور توأم به دو عفونت مبتلا شده بودند. 8/10% کودکان کم وزنی هنگام تولد داشتند و تنها 86% کودکان تغذیه انحصاری با شیر مادر داشتند. در این مطالعه کم وزنی با شیوع بالای هرسه شاخص تغذیه­ای، سابقه عفونت با کوتاه قدی و وضعیت تغدیه نیز با کم وزنی و لاغری ارتباط معناداری را نشان دادند (05/0P<). نتيجه‌گيري: با توجه به نتایج مطالعات قبلی به نظر می­رسد این شاخص­ها رو به بهبودی است ولی برای کاهش شاخص­های سوء تغذیه بهترین روش همان آموزش­های لازم به مادران قبل از تولد و مراقبت از آن­ها برای پیشگیری از تولد نوزادان کم وزن و آموزش مادران در مورد بهداشت خانواده و اهمیت تغذیه با شیر مادر است.

Title: بررسي اپيدميولوژيكي علل و عوامل مرتبط با ناباروري در زوجين نابارور مراجعه¬كننده به درمانگاه بيمارستان پيمانيه جهرم در سال¬هاي 1388- 1378

http://knh.shmu.ac.ir/index.php/site/article/view/984

مقدمه: ناباروري يكي از مشكلات عمده ژنيكولوژي است و به عنوان ناتواني در حاملگي بعد از يك­سال مقاربت، بدون استفاده از هرگونه روش پيشگيري از بارداري تعريف مي­شود. امروزه اطلاعات واقعي و دقيق در مورد ميزان شيوع ناباروري در ايران كم است و به نظر مي­رسد دانستن اين اطلاعات در درك مشكل و طرح­ريزي خدمات مراقبتي مؤثر باشد. مراكز تحقيقاتي مختلف، فراواني هاي متنوعي از علل ناباروري را ذكر كرده­اند كه برخي از اين علل نظير عفونت­ها و بيماري­هاي منتقله از راه جنسي در آفريقا شايع­ترند و ا ز سوي ديگر بعضي فاكتورهاي محيطي نیزبه­عنوان عوامل مؤثر در ناباروري در نظرگرفته مي­شوند. مواد و روش­ها: اين مطالعه توصيفي با پرسش­نامه طراحی شده به­صورت مقطعی بر روي 169 زوج نابارور به روش سرشماري انجام گرفت. جمعیت مورد مطالعه کلیه زوجین نابارور مراجعه­کننده به بیمارستان­ها و درمانگاه­های وابسته به دانشگاه علوم پزشکی جهرم طی سال­های 1378 تا 1388 بوده­اند. میانگین و انحراف معیار متغیرهای کمی و درصد فراوانی متغیرهای کیفی محاسبه و ازآزمون کای دو استفاده و مقدار P کم­تراز 05/0 معنادار درنظر گرفته شد. يافته­ها: دراین مطالعه سن بیماران بین 43- 18 سال با میانگین 4/5± 4/27 و سن همسران آن­ها بین 67- 21 سال با میانگین 4/6 ± 33 و تعداد ميانگين طول مدت نازايي 25/45± 9/67 ماه بود و 4/96درصد زوجين در اولين ازدواجشان دچار نازايي شده بودند. 3/63% خانم­ها مبتلا به هيرسوتيسم و 7/67درصد آن­ها سيكل­هاي نامنظم قاعدگي داشتند و 2/15درصد داراي سابقه فاميلي PCO بودند. نتايج آناليز سيمن در 1/91درصد مردان طبيعي گزارش شده بود. در اين بررسي 5/84% نازايي زوجين از نوع اوليه و 5/15% از نوع ثانويه بود. بیش­ترین داروی مصرفی کلومید با 5/85% و پس از آن HCG و HMG به­ترتیب 2/43% و 2/40% بود. 151 مورد لاپاراسكوپي شده و نتايج لاپاراسكوپي نشان دادکه 5/81% خانم­ها از نظر وضعيت رحم طبيعي و 4/11% داراي اندومتريوز بودند. در 8/22% (38 نفر) لوله­هاي رحمي راست و در 34 نفر (6/20%) لوله­هاي رحمي چپ بسته بود. 9/14% اندومتريوما در تخمدان راست در مقابل 1/17% اندومتريوما در تخمدان چپ، 6/82% PCO تخمدان راست در مقابل 3/83% PCO تخمدان چپ وجود داشت. فقط بین سابقه فامیلی یائسگی زودرس و وجود Pco درتخمدان­ها رابطه آماری معنادار مشاهده شد (001/0=P). نتيجه­گيري: در اين مطالعه اغلب زنان درجاتي از چاقي،همراه با هيرسوتيسم داشته، اغلب داراي سيكل­هاي نامنظم قاعدگي بودند و نيمي از آن­ها وضعيت ترشحات هورموني طبيعي داشتند. لذا طبيعي است كه بيش­ترين تشخيص علت نازايي در نتايج لاپاروسكوپي آن­ها PCO باشد. در 8/16% خانم­ها نتايج HSG و در یک پنجم موارد نتايج لاپاروسكوپي غير طبيعي گزارش شده كه نشان­دهنده چسبندگي در لوله و يا رحم بوده است و چون نازایی با سابقه عمل جراحی قبلی مرتبط نبوده، از علت­هايي كه منجر گرديده در اين منطقه شيوع نازايي ثانويه بيش­تر باشد، احتمالا شیوع بيش­تر عفونت­هاي لگني است.

Title: مهم¬ترین عوامل خطر مرگ کودکان زیر یک¬سال کدامند؟

http://knh.shmu.ac.ir/index.php/site/article/view/985

مقدمه: میزان مرگ­ومیر کودکان زیر یک­سال یکی از شاخص­های مهم تعیین­کننده وضعیت سلامت یک جامعه محسوب می­شود و این شاخص تحت تأثیر عوامل گوناگونی قرار دارد. این تحقیق جهت بررسی عوامل دخیل در مرگ­ومیر کودکان زیر یکسال در شهرستان شاهرود اجرا شده است. مواد و روش­ها: این مطالعه بصورت مورد – شاهدی همسان­سازی شده صورت گرفت. مرگ کودک زیر یک­سال در طی سال­های 86 لغایت 87 به­عنوان مورد در نظر گرفته شده و برای هر مورد دو شاهد با همسان­سازی زمان و مکان تولد انتخاب شدند (117 مورد و 234 شاهد). فرم جمع­آوری داده­ها برای هر دو گروه تکمیل شد و سپس اطلاعات حاصله در رایانه ثبت و با نرم­افزار آماری SPSS نسخه 16 و با استفاده از روش آنالیز آماری رگرسیون لجستیک شرطی بصورت تک متغیره و چند متغیره مورد تجزیه و تحلیل قرار گرفت. يافته­ها: بر اساس نتایج آنالیز تک متغیره سن حاملگی کم­تر از 37 هفته (Odds Ratio=9.85)، وزن بدو تولد زیر 2500 گرم (Odds Ratio=15.14) و حاملگی پر خطر (Odds Ratio=3.13) رابطه معناداری با مرگ کودکان زیر یک­سال داشته­اند (P Value<0.05). نهایتاً در آنالیز چند متغیره، دو متغیر وزن زیر 2500 گرم هنگام تولد (Odds Ratio=8.04) و سن حاملگی زیر 37 هفته (Odds Ratio=3.51) رابطه معناداری با مرگ کودک زیر یک­سال نشان دادند. نتیجه­گیری: بر اساس نتایج این مطالعه مشخص شد که دو متغیر وزن بدو تولد زیر 2500 گرم و سن حاملگی زیر 37 هفته مهم­ترین متغیرهایی هستند که شانس مرگ کودک زیر یک­سال را افزایش می­دهند.

Title: بررسي عوامل خطر مرگ کودکان 1 تا 59 ماهه، يک مطالعه مورد- شاهدي لانه گزيده به¬منظور معرفی الگوی بهره¬گیری از اطلاعات داده¬های جاری در سیستم سلامت کشور

http://knh.shmu.ac.ir/index.php/site/article/view/986

مقدمه: هدف مطالعه، ارزیابی علل مرگ زودرس کودکان 1 تا 59 ماهه ساکن مناطق روستائی شهرستان شاهرود می­باشد. مواد و روش­ها: یک مطالعه مورد- شاهد لانه گزیده است. همگروه تولد شامل 10910 متولد زنده که بین سال­های 1378 تا 1387 در مناطق روستائی شاهرود متولد شده­اند می­باشد. موارد (Cases) تمامی کودکان فوت شده در همگروه (65 مورد) بوده­اند. محققین برای انتخاب گروه شاهد، از روش نمونه­گیری Risk set استفاده کرده، به ازای هر مورد، 2 شاهد همسان شده از نظر سن برحسب ماه (جمعاً 130 نفر شاهد) انتخاب شدند. به­منظور تحلیل داده­ها از روش­های رگرسیون لجستیک کاندیشنال (Unadjusted and Adjusted) با کمک نرم­افزار STATA 9.1 استفاده شد. یافته­ها: بین "طول مدت تغذیه با شیر مادر" و مرگ زودرس کودکان (Adjusted OR: 0.856, CI: 0.79-0.93, P= 0.000) هم­چنین بین "تعداد مراقبت­های بهداشتی کودکان" و مرگ زودرس کودکان (Adjusted OR: 0.900, CI: 0.83-0.98, P= 0.017) ارتباط آماری معناداری شناسايی شد. بین "کم وزنی نوزاد هنگام تولد" و مرگ زودرس کودکان نیز یک ارتباط آماری معنادار بینابینی مشاهده گردید (Adjuster OR: 7.380, CI: 1.00-54.41, P= 0.050). نتیجه­گیری: عوامل متعدد و پیچیده­ای به مرگ­ومیر کودکان 1 تا 59 ماهه منجر می­شوند. طراحی و اجرای برنامه­های ترکیبی برای کارکنان بهداشتی و والدین، به ویژه برنامه­های همراه با آموزش تئوری و عملی، در زمینه­های اصلاح تغذیه دوران بارداری، اهمیت شیردهی و هم­چنین توجه به مراقبت­های بهداشتی به هنگام و با کیفیت برای کودکان ممکن است در کاهش مرگ­ومیر زودرس کودکان 1 تا 59 ماهه مؤثر باشد.

Title: بررسی وضع تغذیه دختران نوجوان شهر شاهرود

http://knh.shmu.ac.ir/index.php/site/article/view/987

مقدمه: نوجواني يكي از بحراني­ترين دوره­هاي رشد انسان محسوب مي­شود ميزان و سرعت آن در مناطق مختلف جهان متفاوت است لذا توجه به وضع تغذيه دختران كه در آينده مادران سالمي باشند و بچه­هاي سالمي به دنيا بياورند اهميت دارد. مواد و روش­ها: اين مطالعه به­صورت مقطعي و توصيفي تحليلي با استفاده از روش­هاي مصاحبه و اندازه­های تن­سنجی بر روي 418 دانش­آموز دختر مقطع راهنمايي به روش نمونه­گيري خوشه­اي دو مرحله­اي در سال 1386 انجام گردید: تجزيه و تحليل داده­هاي پژوهش با استفاده از نرم­افزار آماري SPSS مورد تجزيه و تحليل قرار گرفت. يافته­ها: در اين مطالعه 418 دانش­آموز دختر شركت كردند.373 نفر (2/89%) دانش­آموزان در مدارس دولتي و بقيه (8/10%) در مدارس غير انتفاعي درس مي­خواندند. براساس طبقه­بندی BMI و مقایسه آن با استاندارد (9/12%) لاغر (7/12%) در معرض خطر افزایش وزن و (7/1%) چاق می­باشند که با بعد خانوار ارتباط معناداری دارد. از نظر سوء تغذیه زمان گذشته (2/8%)، سوء تغذیه زمان حال و گذشته (5/6%) از سوء تغذیه شدید رنج می­برند. از نظر ميزان مصرف شير 4/35% بين 3-2 بار در هفته شير مصرف مي­كنند و 8/71% از ميان وعده در مدرسه مصرف كرده و بيش­ترين نوع ميان وعده مصرفي نان و پنير (38%) مي­باشد (8/56%) از دختران نوجوان صبحانه مصرف می­کنند. میزان دریافت کلسیم و آهن نسبت به استاندارد کم­تر بوده است. نتيجه­گيري: دوره نوجواني به خاطر رشد سريع نياز به مواد مغذي دارد از طرف ديگر به­دليل افزايش نياز به انرژي در اين دوران نياز به ويتامين­هاي گروه B كمپلكس كه در متابوليسم انرژي مؤثر هستند مورد نياز است. از طرف ديگر ناز به آهن و كلسيم وجود دارد در حين جهش رشد سريع در نو جوان نياز به آهن افزايش مي­يابد افزايش نياز ارتباطي بازمان و اندازه جهش رشدي و نيز بلوغ جنسي و شروع قاعدگي داردمطالعه روي ارزش بيولوژيكي آهن رژيم غذايي. با توجه به نتايج به­دست آمده از اين تحقيق براي بهبود وضعيت نسل آينده به خصوص دختران كه مادرن فردا هستند بايد نهايت تلاش خود را براي افزايش آگاهي­هاي تغذيه­اي خانواده­ها به خصوص مادران بكنيم تا شاهد جامعه­اي سالم و پويا در آينده باشيم و لذا لزوم پايش و ارايه خدمات تغذيه­اي براي اين گروه سني به­صورت فردي و گروهي توصيه مي­شود.

Title: مقايسه كارايي مدل رگرسيون دو جمله¬اي منفي و مدل رگرسيون پواسني در تحليل داده¬هاي باروري

http://knh.shmu.ac.ir/index.php/site/article/view/988

مقدمه: مدل‌بندي آماري تغييرات مشاهده شده در داده­ها را از طريق معادلات رياضي تبيين مي‌‌نمايد. در حا‌لتي كه متغير پاسخ (وابسته)،گسسته باشد مدل پواسن مورد استفاده قرار مي­گيردو در صورتي كه شرايط مدل پواسن برقرار نباشد، بهتر است از مدل­هاي دو جمله‌اي منفي استفاده كرد. لذا هدف از اين مطالعه تأكيد بر توجه به ساختار داده­ها، معرفي مدل دوجمله­اي منفي و به­كار بردن اين مدل جهت برآورد ضرايب عوامل مؤثر بر تعداد فرزندان و مقايسه آن با نتايج مدل رگرسيون پواسن معمولي است. مواد و روش­ها: در اين مطالعه ضمن معرفي مدل دو جمله­اي منفي،كاربرد آن در تحليل داده­هاي باروري به­كار رفته است. اين داده­ها از يك نمونه 1019 نفري زنان روستايي استان فارس به­صورت مقطعي و با استفاده از روش نمونه­گيري طبقه­بندي به­دست آمد. متغير تعداد فرزند آن زنده متولد شده يك زن به­عنوان متغير پاسخ شمارشي جهت كاربرد مدل در نظر گرفته شده است. سطح معناداري 5 درصد در نظر گرفته شد. يافته­ها: در مجموع 1019زن روستايي مورد بررسي قرار گرفتند كه ميانگين فرزندان هر زن 3/4 و واريانس آن 3/8 بود. مقدار آماره آزمون،   log-likelihoodبراي مدل پواسني معمولي 93/1950- وبراي مدل دو جمله­اي منفي 93/1946- بود. نتيجه­گيري: نتايج نشان دادكه داده­ها Over Dispersionدارد و بر اساس معيار­هاي انتخاب بهترين مدل، مدل دو جمله‌اي منفي و يا تعميم يافته آن جهت تحليل اين داده­ها مناسب است و مي­تواند ضرايب عوامل مؤثر بر تعداد فرزند را دقيق­تر برآورد نمايد.

Title: شناسایی و رتبه¬بندی عوامل مؤثر بر کیفیت خدمات طرح پزشک خانواده با استفاده از مدل کانو در شهرستان کاشان سال 1388

http://knh.shmu.ac.ir/index.php/site/article/view/989

مقدمه: امروزه همگام با پیشرفت تکنولوژی وصنعت،انتظارات انسان­ها به­عنوان مشتریان دریافت­کننده خدمت یا کالا افزایش یافته است بنابراین می­توان گفت که پیشرفت سازمان­ها در گرو همگام شدن با نیازهای در حال تغییر مشتریان و موفقیت در جلب رضایت آن­ها است. یکی از بخش­های دولتی ارايه­دهنده خدمت که بخش مهمی از هزینه­های ملی به آن اختصاص می­یابد بخش خدمات مراقبت سلامت می­باشد. بنابراین ضروری است که کیفیت خدمات این بخش مورد بررسی قرار بگیرد. در این بررسی سعی برآن است تا به وسیله مدل کانو در قالب ابعاد پنج­گانه کیفیت خدمات پاراسورامان عوامل مؤثر برکیفیت خدمات در طرح پزشک خانواده از دیدگاه مشتری شناسایی، رتبه­بندی و ابزاری جهت سنجش کیفیت خدمات این طرح طراحی و ارايه گردد. مواد و روش­ها: پژوهش حاضر یک مطالعه توصیفی تحلیلی است که به روش مقطعی بر روی نمونه­ای به حجم 240 نفر انجام گرفت. برای جمع­آوری اطلاعات از پرسش­نامه استفاده شد و نتایج با استفاده از برنامه آماری SPSS و آماره­های فراوانی مطلق، قاعده تعدیل فراوانی، تعیین درجه بهترین و بدترین حالت برای هر ویژگی و میانگین موزون مورد تجزیه و تحلیل قرارگرفت. یافته­ها: نتایج نشان داد اکثریت مؤلفه­های مورد سنجش در طبقه نیازهای الزامی قرار دارند که از این میان کم­ترین میانگین وزنی در بعد دلسوزی و تعهد (برخورداری پزشک از وجدان کاری، در بعد قابلیت اعتماد، محرمانه ماندن اطلاعات و در بعد اطمینان،دانش به روز و تجربه بالای پزشک) مشاهده گردید. نتیجه­گیری: بر اساس نتایج حاصل از پژوهش در تمامی ابعاد، مؤلفه­های صداقت پزشک، محرمانه باقی ماندن اطلاعات، احساس مسوولیت در زمان بروز مشکل و برخورد مؤدبانه بیش­ترین تأثیر را در تأمین کیفیت خدمات نشان دادند. لذا در طراحی الگوی سنجش کیفیت خدمات طرح پزشک خانواده می­تواند مورد توجه قرار گیرد.

Title: بررسی نظرات متخصصین سلامت در خصوص نقش¬های فعلی و مطلوب پزشکان عمومی در نظام سلامت ایران

http://knh.shmu.ac.ir/index.php/site/article/view/990

مقدمه: فرآیند تعیین نقش، به­عنوان اولین و مهم­ترین گام در فرآیند برنامه­ریزی درسی محسوب شده و تاکنون به­طور سیستماتیک مطالعه­ای در این زمینه انجام نگرفت. در نظام سلامت، با توجه به هرم خدمات سلامت، پزشکان عمومی به­عنوان متولی اصلی نظام مراقبت سلامت اولیه، دارای جایگاه کلیدی می­باشند، لذا تعیین نقش آن­ها در میان پزشکان و پیراپزشکان از اولویت بیش­تری برخوردار می­باشد. مواد و روش­ها: نوع مطالعه، کمی و از نوع پیمایشی- پرسش­نامه­ای است و 40 نفر از متخصصین سلامت، همکاری کردند. انتخاب نمونه به­صورت هدفمند و با روش نمونه­گیری آسان صورت گرفت. برای جمع­آوری داده­ها از پرسش­نامه­ای نیمه ساختارمند و محقق ساخته استفاده شد. تحلیل آماری با نرم­افزار SPSS و استفاده از آزمون مقایسه میانگین­ها و آزمون تعقیبی شفه انجام شد. یافته­ها: 30 % شرکت­کنندگان زن و 70 % مرد بودند و اظهار داشته­اند که در حال حاضر پزشکان عمومی نقش­های، ارايه خدمات درمانی، مدیریت اجرایی مراکز سلامت و رهبری خدمات بالینی و هماهنگ­کنندگی درمان بیماران بر عهده دارند، ولی لازم است به­ترتیب اولویت، نقش­های ارايه خدمات درمانی، رهبری خدمات بالینی و هماهنگ­کنندگی درمان بیماران، ترویج بهداشت، آموزش بیمار و ارتقاء سلامت فردی، مدیریت اجرایی مراکز سلامت و دروازه بان نظام سلامت را بر عهده گیرند. نتیجه­گیری: با توجه به نتایج، شرکت­کنندگان بر این عقیده بودند که درحال حاضر پزشکان عمومی به­طور عمده به عنوان ارايه­کننده خدمات درمانی به بیماران، ایفای نقش می­کنند و لازم است علاوه بر این نقش سنتی، به نقش­های دیگری نیز بپردازند تا نظام سلامت کشور قادر به پاسخگویی به نیازها و انتظارات جامع مشتریان خویش بوده و لذا باید در برنامه درسی آموزش پزشکی عمومی کشور مداخلاتی صورت گیرد.

Title: ميزان انتقال دانش حاصل از پژوهش در طرح¬های دانشگاه علوم پزشکی گلستان طی سال¬های 85 -1383

http://knh.shmu.ac.ir/index.php/site/article/view/991

مقدمه: علی­رغم رشد خیره­کننده انتشارات علمي دو دهه اخير در حوزه علوم پزشکي در ايران، ولی مطالعات انجام شده در ايران نشان مي­دهند خلا در بعد به­كارگيري نتايج دانش می­باشد. لذا این مطالعه به­منظور بررسی میزان انتقال دانش حاصل از پژوهش در طرح­های دانشگاه علوم پزشکی گلستان صورت پذیرفت. مواد و روش­ها: کليه طرح­هاي تحقيقاتي دانشگاه علوم پزشکي گلستان طي سال 85-1383 که تا نيمه دوم سال 1387 گزار ش نهايي آن­ها ارايه گرديده بود مورد بررسی قرار گرفتند. ابزارهاي گردآوري داده­ها شامل فرم گردآوري داده­هاي طرح و پرسش­نامه پژوهشگر بود. يافته­ها: مرور 102 پيشنهاد پژوهشي نشان داد كه مجموع هزينه طرح­هاي مورد بررسي كمي بيش­تر از 3 ميليارد ريال بود. در 6 طرح (8/5%) براي فعاليت­هاي انتقال دانش حاصل از پژوهش هزينه­اي در نظر گرفته شده بود. در زمينه فعاليت­هاي غير فعال انتقال دانش، در پژوهش­هاي باليني و سيستم بهداشتي طبق نظر محققين سهم فعاليت انتشار نتايج در مجلات داخلي و بين المللي بيش­تر از ساير فعاليت­ها بوده در حالي­كه در پژوهش­هاي علوم پايه يافته­هاي پژوهش بيش­تر در كنفرانس­ها و سمينارها و همايش­ها انتشار يافته است. در زمينه فعاليت­هاي فعال انتقال دانش، ميانگين امتياز عملکرد پژوهش­گران تنها 9 درصد از کل امتياز ممکن در اين زمينه بود. نتيجه­گيری: مطالعه حاضر نشان داد كه هزينه­هاي در نظر گرفته شده براي فعاليت­هاي انتقال دانش كم مي­باشد كه در اين مورد ايجاد انگيزه در محققين و آموزش محققين در زمينه انتقال پژوهش ضروري به­نظر مي­رسد.

Title: روند شاخص توده بدنی در 15 سال اخير در ايران؛ مرور سيستماتيک و متا آناليز

http://knh.shmu.ac.ir/index.php/site/article/view/992

مقدمه: امروزه اپيدمی چاقی در جهان در حال وقوع است به­طوري­که بيش­تر باربيماری مربوط به آن مربوط به کشورهای درحال توسعه است. يکی از شاخص­های مهم جهت بررسی وضعيت آن در جوامع، بررسی روند تغييرات شاخص توده بدنی (BMI) است. اين مطالعه ثانويه جهت بررسی اين روند در طی سالهای اخير به تفکيک گروه­های جنسی و سنی در ايران طراحی و اجرا شده است. مواد و روش­ها: اين مطالعه با روش مرور سيستماتيک، مطالعات قبلی و گزارشات چاپ شده و در حال چاپ که BMI را در جامعه ايرانی مورد بررسی قرار داده بودند را با استفاده از ابزار مشخص مورد نقد قرار داده و مطالعاتی که از اعتبار کافی برخوردار بودند را وارد مرحله استخراج و آناليز شدند. منبع جستجوی منابع، بانک­های اطلاعاتی خارجی (Medline و EMBASE) و داخلی (Iranmedex، SID، INL، Irandoc) و گزارشات وزارت بهداشت بود. با استفاده از دستورات Meta در نرم­افزار STATA آناليز با مدل تصادفی انجام شد. يافته­ها: نهايتاً 37 مطالعه وارد مرحله آناليز شدند. اينها در مجموع شامل 119298 مرد و 106598 زن بودند. ميانگين BMI در مردان و زنان به­ترتيب (محدوده اطمينان 95%، 4/24 - - 3/25) 8/24 و (محدوده اطمينان 95%، 9/25 -1/27) 5/26 بود (001/0>P). مدل متارگرسيون سن و جنس را به­عنوان دو فاکتور مهم در ايجاد هتروژنيتی در BMI افراد معرفی کرد. ميانگين BMI در مردان از kg/m2 2/22 در گروه سنی زير 20 سال تا kg/m2 4/25 در دهه ششم زندگی افزايش می­يابد. ميانگين BMI در زنان در همه گروه­های سنی از مردان همگروه بيش­تر است. روند افزايشی در مردان در دهه سوم زندگی ثابت شده در حالي­که در زنان اين روند همچنان افزايش دارد. روند تغييرات BMI در چند سال اخير در زنان هميشه بيش­تر از مردان بوده است. نتيجه­گيری: وضعيت چاقی در ايران، به­خصوص در زنان، در محدوده خطر قرار دارد. نسبت به مردان، زنان همواره در خطر بيش­تری برای چاقی قرار داشته­اند. سرعت چاقی در دهه سه و چهار زندگی بيش­تر از ديگر مقاطع عمر است. در طی سال­های اخير مداخلات و برنامه­های پيگيرانه چندان مؤثر نبوده و تغييری در جهت بهبود روند چاقی بوجود نيامده است.

Title: تعیين شیوع پرفشاری خون در جمعیت شهری و روستایی ساکن استان فارس از دیدگاه طرح ملی مراقبت عوامل خطر بیماری¬های غیرواگیر در سال 1385 و 1386

http://knh.shmu.ac.ir/index.php/site/article/view/993

مقدمه: فشار خون بالا به دلیل شیوع بالای آن و همراهی آن با بیماری­های قلبی - عروقی یکی از مشکلات بهداشتی مهم در تمامی کشورهای دنیا به شمار می­رود.. هم­چنین به گفته سازمان جهانی بهداشت، 13% از کل مرگ­های دنیا را به پرفشاری خون نسبت می­دهند. از این رو، هدف این مطالعه بررسی وضعیت فشار خون در افراد 64-15 ساله ساکن استان فارس از دیدگاه طرح ملی مراقبت عوامل خطر بیماری­های غیر واگیر در سال 1385 و 1386 بوده است. مواد و روش­ها: در این مطالعه که به­صورت مقطعی انجام شده است، مجموعاً 2000 نفر از گروه­های سنی 15 تا 64 ساله که شامل 50 خوشه 20 نفره برای هر سال (85 و 86) بوده است، با استفاده از روش نمونه­گیری خوشه­ای، به­عنوان جمعیت مورد مطالعه تعیین گردیدند. پس از شناسایی افراد مورد نظر، در روز معینی به درب خانه ایشان مراجعه شد و پرسش­نامه­های مربوطه برای آن­ها تکمیل گردید، ونهایتاً با استفاده از نرم­افزار آماری SPSS مورد تجزیه و تحلیل قرار گرفت. یافته­ها: در سال 1385، 4/15 درصد مردان، 13 درصد زنان و 2/14 درصد از کل جمعیت تحت مطالعه دارای فشار خون بالا (سیستول بالای 140 و یا دیاستول بالای 90) بودند، این در حالی است که در سال 1386 این مقدار افزایش پیدا کرده، به­طوری که فشار خون بالا در بین مردان 4/27 درصد، زنان 8/21 درصد و در کل جمعیت تحت مطالعه طی سال 1386 این مقدار به 6/24 درصد رسیده است. بیش­ترین شیوع پر فشاری خون نیز در گروه سنی 64-55 ساله مشاهده شد. نتیجه­گیری: بر اساس نتایج این مطالعه، افزایش روند پرفشاری خون در سال 86 نسبت به سال 85 مشهود بوده است (2/14% در مقابل 6/24%). از آنجايی­که یکی از عوامل خطر بیماری­های قلبی و عروقی، افزایش فشار خون می­باشد و به دلیل افزایش روند مرگ­ومیر ناشی از بیماری­های غیرواگیر به­خصوص در کشورهای در حال توسعه و با توجه به اعلام سازمان جهانی بهداشت مبنی بر در اولویت قرار دادن پیشگیری از بیماری­های غیرواگیر از طریق کاهش عوامل خطر لازم است تا مسوولان بیش از پیش در این زمینه فعالیت نمایند.

Title: برآورد اندازه جمعيت گروه¬هاي پر خطر HIV در مردان 18 تا 45 ساله شهر کرمان با استفاده از روش Network scale up

http://knh.shmu.ac.ir/index.php/site/article/view/994

مقدمه: تخمين جمعيت گروه­هاي پرخطر يکي مهم­ترين پارامترهاي مورد نياز براي برنامه‏ريزي­هاي کلان کشوري در زمينه بيماري HIV/AIDS است. به­دليل مخفي بودن و دشواري در يافتن افراد متعلق به اين گروه­ها بايست به روش­هاي غير مستقيم تخميني از جمعيت اين گروه­ها زد و در اين بين روش network scale up يکي از روش­هاي مؤثر و متداول مي­باشد. هدف از انجام اين مطالعه برآورد اندازه گروه­هاي پرخطر HIV در مردان 18 تا 45 ساله کرماني با استفاده از اين روش است. مواد و روش­ها: براي تخمين تعداد افراد پر خطر HIV، نمونه­اي به اندازه 500 نفر از مردان جوان 18 تا 45 ساله شهر کرمان انتخاب شد. براي جمع‏آوري اطلاعات از پرسش­نامه استفاده شد که شامل 1) از اين افراد پرسيده شده بود که چند مرد 18 تا 45 ساله کرماني را بر اساس معيار‏هاي تعريف شده براي براي تخمين اندازه شبکه مي‏شناسند،2) در مورد گروه­هاي پرخطر (مصرف‏کننده موادمخدر، مصرف موارد روانگردان، مصرف موارد به­صورت نامشخص و مواد الکلي) از افراد پرسيده شد که در شبکه خود آيا کسي را مي‏شناسند که متعلق به اين گروه­ها باشند. اگر جواب آنها بلي بود از آن­ها سؤال مي‏شد که در هر يک از اين گروه­ها چند نفر را مي‏شناسند. يافته­ها: برآورد اندازه جمعيت بر اساس ميانگين ساده تعداد افراد شناخته شده در گروه­هاي پرخطر مصرف‏کننده مواد مخدر، مصرف موارد روانگردان، مصرف موارد به­صورت نامشخص و مواد الکلي به ترتيب برابر 10402، 2418، 3056 و 22572 نفر بوده است. اما بر اساس 5% ميانگين محصور شده اين اعداد براي اين گروه­ها به ترتيب برابر 6307، 581، 501 و 11143 نفر بوده است. نتيجه­گيري: در صورتي که پيش‏فرض‏هاي اين روش به خوبي برقرار باشد مي‏توان به اندازه‏اي که از روش به دست مي‏آيد اعتماد کرد. اين مطالعه به­صورت يک مطالعه مقدماتي (Pilot) براي اولين بار در ايران انجام گرفته است. براي برآورد اندازه جمعيت گروه­هاي پرخطر در ايران، پيشنهاد مي‏شود مطالعات جامع‏تري از جمله مطالعات در بين زنان و در گروه­هاي سني متفاوت نيز اجرا شود.

Title: مقایسه طرح¬های تنها موردی و مورد شاهدی در ارزیابی اثر متقابل ژن- محیط با استفاده از داده¬های سرطان پستان

http://knh.shmu.ac.ir/index.php/site/article/view/995

مقدمه: نقش متقابل عوامل ژنتیکی و محیطی در علم اپیدمیولوژی به­طور گسترده­ای مورد توجه قرار گرفته است. روش­های سنتی مطالعه اثرات متقابل عوامل ژنتیک و محیط نیازمند گروه شاهد می­باشد و انتخاب گروه شاهد مناسب برخی مشکلات را به همراه دارد، لذا روش­های نوینی برای مطالعه این اثرات به وجود آمده­اند که از جمله این روش­ها، روش تنها موردی می­باشد. در این روش فقط از داده­های موارد استفاده می­شود. در این تحقیق ما برآنیم تا بین دو طرح مورد شاهدی و تنها موردی مقایسه­ای انجام دهیم که برای این مقایسه از داده­های بیماران مبتلا به سرطان پستان استفاده نمودیم. مواد و روش­ها: این مطالعه با استفاده از دو طرح تنها موردی و مورد شاهدی همسان شده انجام گرفته است. جمعیت تحت مطالعه شامل زنان با سابقه سکونت 5 سال و بیش­تر در شهر شیراز می­باشد. 300 مورد مبتلا به سرطان پستان که در هر دو طرح از آن استفاده شده است با روش تصادفی از میان پرونده­های موجود در کلینیک پستان در درمانگاه شهید مطهری انتخاب شدند. در این مطالعه گروه شاهد مورد استفاده در طرح مورد شاهدی همسان شده از میان زنان مراجعه­کننده به درمانگاه شهید مطهری استفاده شده است. ما اثرات متقابل ژن- محیط را مورد بررسی قرار دادیم. در این مطالعه از سابقه سرطان پستان در بستگان در جه یک به­عنوان عامل نماینده ژنتیک استفاده گردید و کارایی مطالعه با مقایسه حدود اطمینان، خطای معیار، P-value و Log likelihood انجام گردید. يافته­ها: قبل از انجام طرح تنها موردی فرض استقلال ژن محیط با استفاده از تکنیک استاندارد آماری چند متغیره ما بین هر یک از عوامل محیطی و سابقه خانوادگی سرطان پستان ارزیابی شده و فرض استقلال برای همه متغیرها برقرار بود. با توجه به نتایج به­دست آمده در همه برآوردهای اثر متقابل، کارایی طرح تنها موردی بالاتر از طرح مورد شاهدی بود. حدود های اطمینان و log likelihood در همه متغیرها در طرح تنها موردی بهتر از طرح مورد شاهدی بود. خطای معیار در متغیرهای سن اولین قاعدگی، اولین زایمان در بالای 34 سال و افراد زایمان نکرده، سابقه تولد زنده، سابقه شیردهی و مصرف قرص های پیشگیری از بارداری در طرح تنها موردی کم­تر از طرح مورد شاهدی بود. مقدار P در متغیرهای سن اولین قاعدگی، اولین زایمان در بالای 34 سال و افراد زایمان نکرده، سابقه تولد زنده و سابقه شیردهی در طرح تنها موردی کوچکتر از طرح مورد شاهدی همسان شده بود. نتیجه­گیری: یافته­های این مطالعه نشان داد که در صورت برقراری فرض استقلال، مدل تنها موردی روش کارایی به لحاظ آماری، حجم نمونه و راحتی نسبی اجرا برای مطالعه اثر متقابل می­باشد.

Title: الگوي جغرافيايي ميزان¬هاي ميرايي و اپيدميولوژي سرطان معده در ايران

http://knh.shmu.ac.ir/index.php/site/article/view/996

مقدمه: سرطان معده شايع­ترين سرطان در مردان ايراني مي­باشد. شواهد موجود نشان مي­دهد که ميزان بروز آن در مناطق جغرافيايي کشور تفاوت دارد، با اين حال الگوي بروز سرطان معده در ايران مشخص نيست. يرايي سرطان معده در استان­هاي مختلف کشور پرداختيم. مواد و روش­ها: با استفاده ازداده­هاي ثبت کشوري علل مرگ­ومير، ميزان ميرايي استاندارد شده سني (ASMR) سرطان معده در 29 استان کشور تعيين و ميزان­ها را بر اساس جنس و محل سكونت (شهري و روستايي) محاسبه و طبقه­بندي کرديم. نسبت ميزان­هاي بروزیه ميرايي را در چهار استاني كه ميزان­هاي ميرايي و بروز جمعيتي آن­ها نيز در دسترس بود تخمين زديم. يافته­ها: در سال­هاي 1384-1385 در 29 استان ايران 1000 مورد فوت ناشي از سرطان معده گزارش شده بود. ميزان ميرايي استاندارد شده سني به­ترتيب در ميان مردان و زنان 15 و 1/8 در هر 000/100 نفر مي­باشد. بالاترين ميزان ميرايي در استان كردستان ( ASMR=29.1 به ازاي هر 000/100) و کم­ترين ميزان در استان هرمزگان (ASMR=5 به ازاي هر 000/100) مشاهده شده است. ميزان مرگ­ومير ناشي از سرطان معده در مردها دو برابر زن­ها و در روستاها دو برابر شهر نشينان بود. ميانگين ميزان بروز به ميزان مرگ­ومير سرطان معده درمردان 3/2 و در زنها 9/1 بود.. نتيجه­گيري: کشور ما از نظر ميزان بروز و مرگ­ومير سرطان معده جزو مناطق پر خطر محسوب مي­شود. البته ميزان­ها در مناطق مختلف کشور تفاوت زيادي با هم دارند که براي يافتن دلايل آن تحقيقات بيش­ترضروت دارد. نتايج اين مطالعه براي تدوين برنامه جامع كنترل سرطان معده و کاهش بار اين بيماري در کشور راهگشا مي­باشد.

Title: اپیدمیولوژی بیماری تولارمی و نقش آن در بيوتروريسم

http://knh.shmu.ac.ir/index.php/site/article/view/997

بیماری تولارمی یکی از بیماری­های مشترک بین انسان و حیوان است که به نام­های مختلف از قبیل تب خرگوش (Rabbit Fiver)، تب دی فلای (Dee-Fly Fever)، بیماری اوهارا (Ohara Disease ) نامیده می­شود. عامل بیماری فرانسیسلا تولارنسیس می­باشد. تمام سوش­های آن به لحاظ سرولوژیکی یکسان ولی به لحاظ اپیدمیولوژیکی وبیو شیمیایی به دوگروه بیولوژیک تیپ A (بیووارتولارنسیس) و تیپ B (بیووارپالااریکتیکا) تقسیم می­شوند. وسعت انتشار آن با مخازن حیوانی مختلف می­باشد. نشانه­های بالینی آن بر حسب راه ورود عامل عفونی به بدن و حدت بیماری­زائيی سوش­های مختلف آن متفاوت و به اشکال تنفسی، تیفوئیدی، چشمی، غده­ای و احشایی دیده می­شود. به لحاظ اپیدمیولوژی این بیماری در سال 1911 به­عنوان بیماری شبه طاعون جوندگان توصیف و سپس به­عنوان یک بیماری بالقوه شدید و کشنده در انسان مطرح شد. اپیدمی­های آن در دهه­های 1930 و 1940 به­دنبال مصرف آب آلوده در اروپا و شوروی سابق درانسان و نوع حیوانی آن در ایالات متحده به وقوع پیوست. بزرگ­ترین طغیان ثبت شده ناشی از تولارمی استنشاقی در سال 1967- 1966 در یک منطقه کشاورزی در سوئد بوده است. این بیماری در تمام قسمت­های آمریکای شمالی و نواحی زیادی از اروپا، شوروی سابق و چین و ژاپن شایع است. تشخیص سرولوژیک حیوانی این بیماری برای نخستین بار در ایران در سال 1973 توسط محققین بخش اپیدمیولوژی انستیتوپاستور ایران انجام پذیرفت. هم­چنین در سال 1360 اولین مورد تولارمی غده­ای در انسان توسط دکتر یونس کریمی رئيس اسبق بخش اپيدميولوژي تشخیص داده شد.بیماری تولارمی می­تواند برای مقاصد جنگ میکروبی و بیوتروریسمی به­صورت انتشار افشانه­ای به­کار گرفته شود. از طرفی دیگر به-دلیل اینکه افتراق پنومونی تنفسی آن از طغیان طبیعی بیماری­های رایج به­ویژه آنفلوآنزا و پنومونی­های آتیپیکال و یا یکی از انواع پنومونی­های ایجاد شده به­وسیله جنگ افزارهای بیولوژیک نظیر طاعون، آنتراکس یا تب کیو کار مشکلی خواهد بود. در نتیجه با عنایت به اهمیت موضوع لزوم مطالعات اپیدمیولوژیک به­ویژه تشخیص سریع آن در مراکز مختلف به­عنوان یکی از اهداف و پیشنهادات مهم در این كنگره مطرح می­گردد.

Title: تبیین پارامتر¬های مورد نیاز برای مدل¬سازی شدت انتقال ویروس اچ¬آی¬وی در کشور در گروه¬های در معرض خطر

http://knh.shmu.ac.ir/index.php/site/article/view/998

مقدمه: کنترل اچ­آی­وی، برنامه­ریزی بر­مبنای گروه­های پرخطر را می­طلبد. به دلیل نقایص اطلاعاتی، برآورد­های مستقیم شدت آلودگی و سرعت سرایت اچ­آی­وی در دنیا و ایران مقدور نیست. بهترین راه، روش­های مد­لسازی است. یکی از برنامه­های توصیه­شده UNAIDS/WHO برای مد­لسازی اچ­آی­وی، MOT (mode of transmission) می­باشد که می­تواند بر اساس توزیع فعلی عفونت و الگوی خطر در زیر­گروه­ها، تعداد مورد انتظار عفونت اچ­آی­وی درآینده را مدل­سازی نماید. (http://data.unaids.org/pub/Manual/2007/20090407_ModeOfTransmission_manual2007 _en.pdf). برنامه MOT برای انجام مد­لسازی به چندین پارامتر(جمعیت، رفتار­های پر­خطر و شیوع اچ آی وی در زیر­گروه­ها) نیازدارد و لازم است به کمک مرور سیستماتیک، اطلاعات هر­کدام ازاین پارامتر­ها را برآورد نمود. مواد و روش­ها: برای کسب اطلاعات در­مورد پارامتر­های مورد نیازMOT، به مرور ساختارمند پایگاه­های اطلاعاتی مهم (Pubmed, IranMedex, EmroMedix and SID)پرداخته و همچنین گزارشات وزارت بهداشت و دیگر سازمان­ها جمع­آوری شد. درنهایت، با افراد کلیدی در بخش­های مختلف که با زیر­گروه­ها در تماس بودند، ارتیاط برقرار شد تا حداکثر اطلاعات کسب گردد. یافته­ها: در­آینده نزدیک مهم­ترین گروه­های پرخطر مورد­توجه در برنامه، زنان تن­فروش و مشتریان آن­ها، مصرف­کنندگان تزریقی مواد، و مردان دارای تماس جنسی با سایر مردان، می­باشند. مهم­ترین پارامتر­های مورد­نیاز برای همه این گروه­ها، اندازه جمعیت بوده که در مستندات موجود فقط تخمینی از جمعیت مصرف­کنندگان تزریقی به­دست­آمد (در حدود 250000-200000). البته دقت و شیوه برآورد علمی این عدد روشن نیست. در­مورد رفتار­های پرخطر مصرف­کنندگان تزریقی مانند میزان تزریقات مشترک و غیر­ایمن(در حدود 30%) و رفتار­های جنسی (روابط جنسی خارج از ازدواج در حدود 38%) اطلاعات تا­حدی قابل قبول وجود دارد ولی اطلاعات سایر گروه­های پرخطر بخصوص در­مورد مردان دارای تماس جنسی با سایر مردان، محدود و تقریباً در­حد صفر می­باشد. نتیجه­گیری: برنامه MOT اطلاعات کاربردی فراوانی را برای سیاستگزاران فراهم می­نماید. البته دقت این یافته­ها بستگی به میزان دسترسی و دقت مقادیر پارامتر­های مورد نیاز این برنامه دارد. در­حال حاضر مستندات جامع برای کسب چنین اطلاعاتی وجود ندارد، انجام مطالعاتی برای دست­یافتن به این پارامتر­ها ضروری به نظر می­رسد.

Title: مدل¬سازی تأثیر تغییر هرم سنی و جنسی بر بار بیماری¬های قلبی عروقی طی دو دهه آینده در ایران

http://knh.shmu.ac.ir/index.php/site/article/view/999

مقدمه: سهم عمده بار بیماری­ها در جهان به بیماری­های قلبی عروقی نسبت داده می­شود. در این مطالعه با استفاده از مدل­سازی، تأثیر تغییرات هرم سنی و جنسی بر میزان مرگ و بار ناشی از بیماری­های قلبی عروقی طی دو دهه آینده را پیش­بینی می¬کنیم. مواد و روش­ها: اطلاعات مورد نیاز با استفاده از مرور ساختار یافته در منابع معتبر داخل و خارج کشور استخراج و بر اساس این اطلاعات هرم سنی و جنسی جامعه (ایران) برای 20 سال آینده پیش¬بینی گردید. جهت برآورد بار ناشی از این بیماری­ها درگروه­های سنی و به تفکیک جنس از مدل رگرسیون خطی استفاده شد. به­منظور اطمینان از اعتبار مدل، معادله به­دست آمده برای نمونه­های دیگری از جامعه تحت مطالعه اجرا گردید. يافته­ها: میانگین سنی جامعه از 4/27 سال در سال 2005 به 34 سال در سال 2025 افزایش می¬یابد. بار ناشی از بیماری تحت مطالعه در سال 2005، 860000 سال عمر از دست رفته و در سال 2025 این رقم به 1600000 سال عمر از دست رفته خواهد رسید، تفاوت بار بیماری­های قلبی عروقی که در سال­های پایه در مردان بیش از زنان بوده در سال 2025 مشهود نیست. بار بیماری در سال 2005 برای مردان و زنان به ترتیب: 445041، 415456 سال و در سال 2025 این ارقام به ترتیب عبارتند از 84368 856521. البته باید به این نکته توجه داشت که این نتایج با فرض ثابت بودن شیوع عوامل خطر و عوامل محافظتی و صرفاً به دلیل افزایش میانگین سنی جامعه روی می­دهد نتیجه­گیری: با توجه به ادامه روند افزایش میزان مرگ ناشی از این بیماری­ها طرح برنامه­های پیشگیری که نسبت به برنامه­های درمانی و نوتوانی هزینه کم­تر و اثر بخشی بیش­تری دارند ضروری به نظر می­رسد. با توجه به مشترک بودن عوامل خطر بیماری­های قلبی عروقی با بسیاری از بیماری­های مزمن دیگر با انجام اینگونه برنامه­ها سود بیش­تری عاید جامعه خواهد شد.

Title: گذار اپیدمیولوژیک و تغییر سیمای سلامت

http://knh.shmu.ac.ir/index.php/site/article/view/1000

گذار اپيدميولوژيك با تغییر الگوی علل مرگ­ومیر و بیماری توأمان با گذار دموگرافیک و تغییرات حاصل از آن در ترکیب سنی جمعیت واقعيتي به نام تغییر سیمای سلامت را بدنبال داشته است. به­طور مشخص تاكنون جامعه جهانی با سه گذار اپيدميولوژيك عمده مواجه بوده، به­طوري­كه اولين مرحله گذار، در اواخر دوره پارينه سنگي (Paleolithic) و دوران نوسنگي (Neolithic)، حادث گرديده و مشخصه بارز این دوره بروز و شیوع بالای بیماری­های عفونی و فوق­العاده مسری و متعاقب آن مرگ­ومیر بسیار زیاد ناشی از این عوامل بوده است و هم­چنین جنگ، قحطی و خشکسالی از مهم­ترین عوامل تعیین­کننده مرگ و بیماری در جامعه جهانی محسوب می­شدند. دومين گذار، با آغاز انقلاب صنعتي شكل گرفت و مشخصات این دوره عبارتند از کاهش بیماری­های عفونی به دنبال بهبود شرایط زندگی و تأمین تسهیلات بهداشتی و کشف برخی واکسن­ها و آنتی­بیوتیک­ها که به پیشگیری و کنترل بیماری­های عفونی کمک شایان توجهی نمود. بالاخره سوّمين گذار كه از حدود سه دهه قبل آغاز گرديده مبتنی بر بروز و شیوع روز افزون بیماری­های مزمن و غیرواگیر می­باشد. در کشور جمهوری اسلامی ایران در یکی دو دهه اخیر شاهد آنیم که با توسعه تکنولوژی و گسترش زیر ساخت­های صنعتی، افزایش شهرنشینی، کنترل نسبی بیماری­های عفونی خصوصاً بیماری­های قابل پیشگیری با واکسن، کاهش میزان­های مرگ­ومیر کودکان، تغییر ساختار سنی جمعیت و افزایش درصد جمعیت سالمندان کشور و با افزایش امید زندگی و هم­چنین به تبع تأثیرات ناشی از تغییرات و تحولات جهانی و بین­المللی تغییرات گسترده­ای در سبک زندگی و به تبع آن سیمای علل بیماری و مرگ­ومیر مردم کشورمان به وقوع پیوسته است. اکنون شایع­ترین علل مرگ به ترتیب بیماری­های قلبی- عروقی، سوانح و حوادث و سرطان و تومورهاست و از طرفی بیماری­های نوپدید و بازپدید عفونی (ایدز، سل، هپاتیت ویروسی و..) را باید جدی گرفت و هم­چنین آسیب­های روانی- اجتماعی همچون اعتیاد، افسردگی و خودکشی بخش قابل توجهی از بار بیماری­ها را به­خود اختصاص داده­اند. پیشگیری و کنترل عوامل خطری همچون کم تحرکی، عادات نامناسب غذایی، اعتیاد، استعمال دخانیات، آلودگی محیط زیست با آلاینده­های جدید و عدم رعایت اصول ایمنی ترافیک شرایط ویژه­ای ایجاد نموده و این شرایط دشوار و پیچیده برنامه­ریزی­ها و سیاستگذاری­های خاص خود را نیاز دارد. برنامه­های غربالگری بیماری­های مزمن و سرطان­ها و هم­چنین مشاوره بیماری­ها و اختلالات رفتاری و روانی اکنون از اولویت برخوردارند و ارتقای کیفیت زندگی همزمان با افزایش امید زندگی از مهم­ترین دغدغه­های متولیان سلامت جامعه است. پرواضح است که مدیریت این شرایط و کنترل این عوامل مشارکت فعالانه و آگاهانه بخش­های مختلف توسعه را می‌طلبد و به­طور مشخص انتظار آن می‌رود که با شتاب هرچه بیش­تر برنامه­های علمی، آموزشی و هم­چنین اجرایی و عملیاتی ما در سطوح مختلف ستادی و محیطی متناسب با این تحولات و اولویت­ها طراحی و به اجرا گذاشته شود.

Title: مقایسه شاخص‌های مختلف تجویز در نسخ بیمه پزشکان عمومی با پزشکان متخصص استان زنجان در سال 87

http://knh.shmu.ac.ir/index.php/site/article/view/1001

مقدمه: تجویز دارو یکی از مهم­ترین حلقه­های درمان می‌باشد که اگر بعد تشخیص، تجویز درست و یا مناسبی صورت نگیرد ممکن است باعث تحمیل عوارض جانبی و گاه خطراتی به بیمار گردد. مقایسه شاخص‌های مختلف تجویز پزشکان عمومی با پزشکان متخصص تأثیر هر یک بر نتايج کلی استان را نشان می‌دهد که تفاوت­های این دو ناشی از تأثیر آموزش­ها، اطلاع رسانی‌ها و میزان مطالعات پزشکان می‌باشد. هم­چنین مقايسه شاخص­ها نشان می‌دهد که نیازمند به آموزش در چه زمینه­هایی و چه گروه­هایی هستیم. در این مطالعه در مورد تفاوت­های شاخص‌های مختلف تجویز پزشکان عمومی با پزشکان متخصص در استان زنجان در سال 87 بحث و نتیجه‌گیری گردید. مواد و روش­ها: در این مطالعه با استفاده از کلیه نسخ بیمه (شامل تامین اجتماعی و خدمات درمانی که بیش­ترین میزان نسخ را تشکیل می‌دهند)، موارد مختلفی با استفاده از نرم­افزار پردازشگر نسخ از نسخ موجود استان استخراج گردید و مورد تجزيه و تحليل آماري قرار گرفت و بعد مقایسه شدند. يافته­ها: در مجموع 854788 نسخه در سال 87 بررسی شد، که507471 نسخه مربوط به پزشکان عمومی و 240559 نسخه مربوط به پزشکان متخصص (شامل گوش، حلق و بینی 16947، جراحی استخوان و مفاصل 14776، اورولوژی 2311، پوست 6993، چشم 14246، روانپزشکی18897، بیماری­های اعصاب 10724، قلب 12086، جراحی عمومی 19959، زنان 41137، بیماری­های کودکان 52997 و بیماری­های داخلی 33486 نسخه) بود که نتایج ذیل حاصل گردید: 1- بیماران دریافت­کننده داروهای تزریقی تجويزي توسط پزشکان عمومی 55 درصد و توسط متخصصین به ترتیب 25، 51، 17، 9، 1، 8، 33، 6، 43، 19، 14 و 25 درصد و میانگین همه پزشكان استان 43 درصد بود. 2- بیماران دریافت­کننده داروهای آنتی‌میکروبیال تجويزي توسط پزشکان عمومی 54 درصد و توسط متخصصین به­ترتیب 51، 20، 37، 33، 7، 2، 7، 5، 34، 42، 45 و 30 درصد و میانگین همه پزشكان استان 46 درصد بود. 3- بیماران دریافت­کننده داروهای کورتیکواستروئید تجويزي توسط پزشکان عمومی30 درصد و توسط متخصصین به­ترتیب 17، 39، 3، 13، 3، 2، 25، 3، 26، 2، 8 و 16 درصد و میانگین همه پزشكان استان 24 درصد بود. نتیجه‌گیری: پزشکان عمومی همواره درصد بالائی از تجویز داروهای تزریقی، آنتی‌میکروبیال و داروهای کورتیکواستروئید را در مقایسه با آمار كلي استان داشته‌اند و متخصصین در مجموع درصدهاي بهتري در اين موارد داشته‌اند. برخي از نتايج متخصصين از ميانگين كلي استان بالاتر است كه باتوجه به تخصصشان منطقي به­نظر مي‌رسد که در نهايت لزوم آموزش­هاي بيش­تر به پزشكان عمومي و برخي از پزشكان متخصص ضروري بنظر مي‌رسد.

Title: بررسی اثرات کاهش شیوع دیابت و اختلال قند خون ناشتا بر بار قابل انتساب آن به بیماری¬های قلبی عروقی در ایران

http://knh.shmu.ac.ir/index.php/site/article/view/1002

مقدمه: اطلاع از مقدار بار قابل انتساب دیابت و اختلال قند خون ناشتا به بیماری­های قلبی عروقی به­دلیل اهمیت آن در تدوین راه­کارهای پیشگیری و اولویت­بندی مداخلات مربوطه ضروری است. مطالعه حاضر با هدف تعیین اثرات کاهش شیوع اختلال قند خون ناشتا (Impaired Fasting Glucose)، شیوع ابتلای کنونی به دیابت (Known Diabetes Mellitus) و شیوع مبتلایان به دیابت با تشخیص جدید (Newly Diagnosed Diabetes Mellitus (NDM) ) بر بار قابل انتساب آن به بیماری­های قلبی عروقی در ایران در سال 1388 انجام شده است. مواد و روش­ها: به­منظور محاسبه سهم تأثیر بالقوه Potential Impact Fraction)) و درصد بار قابل انتساب بیماری­های قلبی عروقی از متدولوژی ارزیابی خطر مقایسه­ای (Comparative Risk Assessment) سازمان جهانی بهداشت استفاده گردید. داده­های مربوط به شیوع اختلال قند خون ناشتا (Fasting Plasma Glucose [FPG] ≥6.1 and <7.0 mmol/l)، شیوع ابتلای کنونی به دیابت (If they reported having medication treated diabetes) و شیوع مبتلایان به دیابت با تشخیص جدید (FPG ≥7.0 mmol/l) از سومین دوره بررسی کشوری نظام مراقبت عوامل خطر بیماری­های غیرواگیر در سال 1386 استخراج گردید. هم­چنین داده­های مربوط به اندازه اثر (Adjusted Hazard Ratio) عوامل خطر مربوطه از یک مطالعه معتبر انجام شده در ایران گرفته شد. سهم تأثیر بالقوه در دو حالت سطح حداقل خطر تئوریک و امکان­پذیر محاسبه گردید و از رویکرد شبیه­سازی (Monte Carlo simulation-modeling techniques) برای برآورد عدم قطعیت (Uncertainty) درصد بار قابل انتساب استفاده گردید. يافته­ها: با کاهش شیوع مبتلایان به دیابت با تشخیص جدید در زنان از 05/10 درصد به سطح حداقل خطر امکان­پذیر یعنی 5 درصد، 3/4 درصد از کل سال­های از دست رفته تعدیل شده با ناتوانی (DALYs) به­دلیل بیماری­های قلبی عروقی را می­توان کاهش داد. این مقدار برای مردان 9/2 درصد می­باشد. به­طور کلی با کاهش شیوع IFG و NDM به سطح حداقل خطر تئوریک یعنی صفر در کشور، به­ترتیب 7/0 و 1/6 درصد از کل سال­های از دست رفته تعدیل شده با ناتوانی به­دلیل بیماری­های قلبی عروقی در مردان قابل اجتناب خواهد بود. این مقدار برای زنان به­ترتیب 1/1 و 5/8 درصد است. نتیجه­گیری: نتایج بررسی حاضر، اجرای غربالگری کشوری و دوره ای را در بزرگسالان اثربخش پیش­بینی می­نماید. اگرچه در این مطالعه، داده­های مربوط به شیوع دیابت و اختلال قند خون ناشتا و داده­های اندازه اثر مربوطه از منابع بروز شده و ملی گرفته شده است، با این وجود به­منظور تصمیم­سازی بهتر و اولویت­بندی مداخلات پیشنهاد می­گردد گزارش بار قابل انتساب (Attributable Burden) براساس مطالعات بروز شده محاسبه بار بیماری در سطح ملی و به­صورت تعداد سال­های از دست رفته تعدیل شده با ناتوانی بجای درصد بار قابل انتساب انجام گیرد.

Title: تعیین الگوی بهره¬مندی از نظام ارايه خدمت سلامت (بستری) در کشور در سال 1387

http://knh.shmu.ac.ir/index.php/site/article/view/1003

مقدمه: همواره اندازه­گيري عملکردها و در نتيجه کارآيي نظام سلامت به ازاي يک واحد هزينه مورد مناقشه بوده است. شاخص­هايي که با آن­ها عملکرد نظام سلامت را مي­سنجند عبارتند از: سطح سلامتي جامعه و توزيع آن در مناطق و گروه­هاي اجتماعي، سطح پاسخگويي نظام سلامت و توزيع آن در مناطق و گروه­هاي اجتماعي و ميانگين حفاظت از مردم در مقابل هزينه­هاي سلامتي. اين مطالعه با هدف اندازه­گيري نياز مردم به دريافت خدمات سلامت (بستري) در سال 1387،اقدام آن­ها براي دريافت خدمات، بار مراجعه آن­ها، خدمات ارايه شده به آن­ها، زمان و هزينه­هايي که مردم براي دريافت اين خدمات صرف مي­کنند انجام گرديد. مواد و روش­ها: تحقيق در 30 استان کشور انجام گرديد. از هر استان 380 خانواده شهري و 380 خانواده روستايي مورد مطالعه قرار گرفتند. در داخل هر استان تعداد خانوارهاي هر شهرستان بر اساس جمعيت شهري و روستايي هر شهرستان تخصيص يافته و سپس به­صورت تصادفي خانوار مورد نظر انتخاب و کليه افراد آن وارد مطالعه گرديدند. پرسش­نامه‌اي براي افرادي که نياز به خدمت بستري داشتند تکميل گرديد. يافته­ها: در اين طرح از 102 هزار نفر در کل کشور پرسشگري به­عمل آمد که 9597 نفر در يک سال گذشته نياز به خدمات بستري داشتند. 3/50 درصد شهري و 7/49 درصد روستايي بودند. شايع­ترين علت عدم استفاده از بيمه در شهر و روستا بيمه نبودن سرپرست خانوار ذکر گرديد. بيش­ترين علت مراجعه به بيمارستان حوادث تعيين گرديد. اولين دليل عدم اقدام به بستري بي پولي ذکر گرديد. نتيجه­گيري: تحليل­هاي اوليه نتايج اين مطالعه نشان مي­دهد که عوامل متعددي از جمله سکونت در شهر يا روستا، نوع بيمه، نوع بيماري و سطح اقتصادي اجتماعي خانوار در کيفيت دريافت خدمات بستري نقش مهمي دارند.

Title: ارزیابی جامعه به¬منظور شناسایی مشکلات و تدوین برنامه¬هاي عملیاتی ارتقاء سلامت شهر هشترود، تابستان 1388

http://knh.shmu.ac.ir/index.php/site/article/view/1004

مقدمه: نقش ارزیابی جامعه عبارت است از شناسایی عوامل تأثیرگذار درسلامت جامعه و تعیین میزان دسترسی به منابع آن جامعه برای هدف­گیری درست آن عوامل. در ارزیابی جامعه اعضاي جامعه در ایجاد مشارکت­ها، جمع­آوري داده­هاي مربوط به سلامت، تعیین اولویت­هاي سلامت، شناسایی منابع، و طرح­ریزي برنامه­هاي سلامت جامعه نقش اصلی را به عهده دارند. هدف از مطالعه حاضر، ارزیابی جامعه هشترود و تعیین اولویت­هاي سلامت، تشخیص مشکل و برنامه­ریزي عملیاتی سلامت جامعه بود. مواد و روش­ها: این طرح در دو فاز اصلی انجام شد. فاز اول جهت دستیابی و تعیین مشکلات جامعه و اولویت­بندی مشکلات، فاز دوم: انتخاب مشکل اولویت­دار جهت بررسی بیش­تر و ارايه راهکار برای حل آن بود. با تشکیل تیم ارزیابی جامعه، جمع­آوری داده­های اولیه و ثانویه صورت گرفت. داده­های اولیه از طریق برگزاری جلسات بحث متمرکز گروهی و مصاحبه با کارکنان نظام سلامت، افراد کلیدی جامعه و مردم عادی جامعه گردآوری شدند. داده­های ثانویه نیز بعد از ایجاد هماهنگی با سازمان­های مرتبط جمع­آوری گردید. یافته­ها: مشکلات استخراج شده با استفاده از روش Hanlon اولویت­بندی شد که اشتغال، اعتیاد، جمع­آوری و دفع غیربهداشتی زباله­های شهری و... در اولویت قرار داشتند. از این میان، مشکل بیکاری برای تشخیص ابعاد آن در جامعه انتخاب و ازنظر توزیع و عوامل مؤثر بر آن مورد بررسی قرار گرفت. برای این منظور 625 پرسش­نامه از طریق مصاحبه با افراد 15 ساله و بالاتر به روش نمونه­گیری سیستماتیک خوشه­ای تکمیل گردید. نتایج نشان داد که 8/18 درصد افراد بیکار بودند. هم­چنین عوامل مؤثر بر بیکاری و مشخصات افراد بیکار و مهارت­های آن­ها مشخص گردید. نتیجه­گیری: ارزیابی جامعه در نظام شبکه بهداشت و درمان می­تواند با شناسایی نقاط قوت، ضعف و فرصت­های منطقه ابزار مؤثری براي ارتقاء سلامت باشد.

Title: بررسي تأثير طوفان شن بر مراجعه به بيمارستان¬هاي شهر اراك در تير ماه 1388

http://knh.shmu.ac.ir/index.php/site/article/view/1005

مقدمه: در روزهاي 14 تا 16 تيرماه سال 1388 طوفان شن برخاسته از كشورهاي عراق و عربستان بيش از از نيمي از كشور را فراگرفت. يكي از استان­هاي درگير در اين بلاي طبيعي استان مركزي بوده است. هدف از اجراي اين مطالعه بررسي تأثير آلودگي هوا ناشي از طوفان شن بر وضعيت مراجعه به بيمارستان و بستري به علت بيماري­هاي قلبي و ريوي در شهر اراك بوده است. مواد و روش­ها: با استفاده از مدارك موجود در بخش­هاي پذيرش بيمارستان­هاي شهر اراك داده­هاي مربوط به بيماران مراجعه­كننده سرپايي و بستري اين بيمارستان­ها در تاريخ­هاي 14 تا 16 تيرماه 1388 با مدت مشابه آن در يك هفته قبل مورد مقايسه قرار گرفته­اند. رابطه بين آلودگي هوا و بستري به علت بيماري­هاي قلبي و ريوي و نيز مراجعه به بيمارستان با استفاده از رگرسيون پوآسون بررسي گرديد. يافته­ها: در كل تعداد 982 نفر در مدت تحت مطالعه به بيمارستان­هاي شهر اراك مراجعه كرده بودند كه 713 بيمار (72/6%) مربوط به 3 روز داراي آلودگي هوا و 269 بيمار (27/4%) مربوط به سه روز مشابه بدون آلودگي بوده است. خطر نسبي و حدود اطمينان 95% براي بستري به علت بيماري­هاي قلبي، ريوي و مراجعه به درمانگاه سرپايي به­ترتيب 1/22 (0/70- 2/11)، 2/06 (1/11- 3/81) و 1/38 (1/15- 1/52) بوده است. نتيجه­گيري: آموزش كاهش مواجهه مردم با هواي آلوده در زمان بروز اين نوع آلودگي هوا و نيز آماده بودن بخش­هاي داخلي و ريه بيمارستان­ها ضروري است.

Title: تحليل منابع اطلاعاتي موجود سوانح ترافيکي کشور

http://knh.shmu.ac.ir/index.php/site/article/view/1006

مقدمه: هدف اين مطالعه بررسي وضعيت موجود ثبت اطلاعات سوانح ترافيكي در كشور و ارايه پيشنهادهاي كاربردي در جهت اصلاح نظام يكپارچه اطلاعات سوانح ترافيكي در كشور است. مواد و روش­ها: در یک مطالعه توصیفی با استفاده از منابع اطلاعاتي موجود سازمان­هاي دخيل در امر سوانح ترافيكي شناسايي و سپس با مراجعه مستقيم به اين سازمان­ها از جمله پليس راهنمايي و رانندگي، وزارت راه و ترابري، وزرات بهداشت و مراکز تحقيقاتي مثل پژوهشكده حمل و نقل، مركز تحقيقات تروماي سينا، چگونگي ثبت و گردش اطلاعات موجود در خصوص اپيدميولوژي سوانح ترافيکي توسط پرسش­نامه روا و پایای تأيید شده، توسط کارشناسان آموزش دیده جمع­آوري شد. نظام فعلي گزارش ثبت سانحه ‌ترافيكي ‌براساس اطلاعات جمع‌آوري‌ شده‌ توسط‌ منابع مذكور مورد بررسي و تحليل قرار گرفت. یافته­ها: يافته­ها نشان مي‌دهند كه نظام ثبت تحت شبكه فعلي طراحي شده توسط مركز فن­آوري و اطلاعات راهور نيروي انتظامي اصول اوليه و پايه­هاي كافي براي ايجاد نظام ثبت ترافيك كامپيوتري را دارا مي­باشد. ولي به نظر مي­رسد سيستم به­عنوان يك كل در كشور به خوبي و به شكل متمركز مديريت نمي­شود. نظام اطلاعات تحت شبكه به درستي براي سازمان­هاي درگير توضيح داده نشده و يك توافق همگاني براي استفاده از اين سيستم جديد در سطح كلان كشوري بوجود نيامده است. نتیجه­گیری: پيشنهاد مي­شود كه در نظام ثبت اطلاعات تحت شبكه نقش هر يك از بخش­هاي ديگر مثل سازمان راهداري، وزارت راه، مديريت فوريت­هاي پزشكي با تعريف سطح دسترسي متناسب با وظيفه هر يك از اين نهادها ديده شود و مديريت ثبت اطلاعات در هر بخش به خود آن­ها واگذار شود و يكي از نهادهاي اصلي مثل معاونت راهنمايي و رانندگي با همكاري كميسيون راه مسووليت هماهنگی و اعلام آمار كلي را عهده دار شود.

Title: بررسی وضعیت اپیدمیولوژیک حوادث ترافیکی منجر به جرح در جاده تهران- آبعلی در سال 1387به گزارش پلیس راه

http://knh.shmu.ac.ir/index.php/site/article/view/1007

مقدمه: حوادث ترافیکی نخستین علت بار بیماری­ها و دومین علت مرگ­ومیر در سطح کشور محسوب می­گردد. بررسی و تحلیل از وضعیت اپیدمیولوژیک سوانح ترافیکی به­منظور ارتقای کیفیت ارائه خدمات از الویت­های مهم پژوهشی کشور است از طرفی با توجه به وجود انواع ساختارهای جاده­ای در محور تهران- آبعلی و نظر سنجی از کارشناسان پلیس راهنمایی رانندگی این محدوده برای انجام این مطالعه انتخاب شد. مواد و روش­ها: این مطالعه به­روش هم­گروهی آینده­نگر انجام شده است. آسیب­دیدگان سوانح ترافیکی در زمان حادثه در محور تهران- آبعلی، از ابتدای خرداد 1387 به مدت یک سال وارد مطالعه شدند. اطلاعات از طریق تکمیل پرسش­نامه با همکاری پلیس­راه، جمع­آوری شده و در انتها اطلاعات به­دست آمده از پلیس راه گزارش شد. يافته­ها: در طی انجام مطالعه 242 مصدوم به­دنبال رخداد 343 مورد تصادف رانندگی رخ داد که منجر به فوت 17 نفر گردید. از این تعداد 190(51/78%) مرد و مابقی زن بوده­اند. بیش­ترین تعداد مصدومین در گروه سنی 20- 30 سال قرار داشتند. بیش­ترین وسیله نقلیه درگیر نیز سواری شخصی (9/52%) گزارش گردید. 91% از تصادفات در شرایط جوی مناسب و 66% در روز و 34% درصد از موارد در شب اتفاق افتاده­اند. بیش­ترین پراکندگی جغرافیایی محل حادثه ترافیکی جرحی در 11 تا 15 کیلومتری تهران دیده شد. شایع­ترین انواع حوادث ترافیکی برخورد یک وسیله نقلیه با یک وسیله نقلیه دیگر (25%) و شایع­ترین علت رخداد حادثه به­دلیل عدم توجه به جلو (5/43%) گزارش شد. نتيجه­گيري: با توجه به نتایج این تحقیق به­نظر می­رسد لازم است ویژگی­های ذکر شده در برنامه­ریزی­های آتی جهت کاهش و کنترل سوانح ترافیکی مدنظر قرار گیرند.

Title: آنالیز فضایی- زمانی حوادث رانندگی منجر به جرح و فوت در شبکه ترافیکی و جاده¬های منتهی به شهر مقدس مشهد در سال 1385 با استفاده از سامانه¬های مدیریت اطلاعات جغرافیایی ( (GIS

http://knh.shmu.ac.ir/index.php/site/article/view/1008

مقدمه: حوادث و سوانح یکی از مشکلات اساسی بهداشت عمومی و تهدیدکننده عمده سلامت افراد جامعه است و در دهه­های اخیر در کشورهای در حال توسعه در حال افزایش بوده است. بخش عمده­ای از حوادث را تصادفات رانندگی تشکیل می-دهد در این تحقیق ما بر آنیم تا به وسیله ابزار "مدیریت سیستم­های اطلاعات جغرافیایی- GIS"؛ آنالیزهای دقیق­تری علاوه بر آنالیزهای آماری بر روی این حوادث انجام دهیم. مواد و روش­ها: جمع­آوری و تهیه اطلاعات که نقشه اولیه طی درخواست رسمی از "سازمان ترافیک مشهد" برای حدود 27600 تصادف صورت گرفته در سال 1385 تهیه شد. سپس با استفاده از ابزار GIS و روش­های آماری به­ترتیب آنالیزهای فضایی و آماری بر روی اطلاعات خام اولیه صورت گرفت یافته­ها: خلاصه نتایج آماری به شرح زیر است: کل تصادفات در سال 1385 در شهر مشهد به تعداد 26700 فقره که در تقسیم­بندی کلی به 3 دسته تقسیم می­گردد: 1- خسارتی: 23202 فقره (9/86%). 2- جرحی 3436 فقره (9/12%). 3- فوتی 62 فقره (2/0%). هم­چنین با استفاده از GIS، داده­ها را بر روی نقشه­های دیجیتالی ارايه داده و مورد تحلیل قرار خواهیم داد و همراه با آن تجزیه و تحلیل­های اماری مربوطه را با استفاده از قابلیت­های نرم­افزار Arc GIS 9.3 انجام خواهیم داد. نتیجه­گیری: قابلیت GIS در انجام مطالعات اپیدمیولوزیک بسیار زیاد است با استفاده از این تکنیک می­توان در ابتدا به توصیف بیماری­ها و وقایع و به­دنبال ان به تحلیل ان­ها پرداخت. در این مطالعه می­توان نوع وقایع و مکان­های پرخطر را در شهر مشهد شناسایی نمود و به­دنبال ان با کمک گرفتن از متخصصین مربوطه به راهکار مناسب برای کاهش تصادفات رانندگی کاهش رسید.

Title: اپيدميولوژي جراحات ناشي از حوادث ترافيكي در تهران در سال 1386: مطالعه‌اي مبتني بر جمعيت

http://knh.shmu.ac.ir/index.php/site/article/view/1009

مقدمه: روش­هاي متفاوتي براي تعيين مصدوميت‌هاي ترافيكي وجود دارد ليكن هريك با محدوديت‌هايي مواجه هستند. اين مطالعه با استفاده از رويكرد مبتني بر جمعيت كه احتمال كم‌شماري در آن كم است، به ارزيابي اين مشكل سلامت در جمعيت تهران پرداخته است. مواد و روش­ها: 2488 خانوار تهراني كه به­صورت تصادفي انتخاب شده بودند وارد مطالعه شدند. پرسشگران با مراجعه به محل سكونت ايشان پرسش­نامه‌اي را كه حاوي سؤالاتي در مورد نوع و تعداد وسايل نقليه موتوري خانواده، سابقه تصادف و جراحت در سال 1386 و مشخصات دموگرافيك اعضاي خانواده بود را تكميل نمودند. مصاحبه­ها ضبط شده و پس از كنترل كيفيت و تطابق با پروتكل مصاحبه، پاك شدند. يافته­ها: نمونه مورد بررسي شامل 9100 شهروند مي‌شد. 119 مصدوميت و 3 مورد مرگ ناشي از حوادث ترافيكي در نمونه مورد بررسي يافت شد. در سال 1386 ميزان بروز ساليانه كل حوادث ترافيكي در جمعيت مورد مطالعه 1/13 در هزار (95% CI: 10.8 - 15.6) و حوادث ترافيكي كشنده 33 در صدهزار (95% CI: 6.80 - 96.32) بود. ميزان بروز ساليانه مصدوميت ترافيكي براي موتورسواران 95 در هزار بود. نتيجه­گيري: اين مطالعه مبتني بر جمعيت، تعداد مصدوميت‌هاي ترافيكي را حدود 10 برابر بيش­تر از آمار رسمي به دست داد. اين امر نشان مي‌دهد كه در آمار رسمي، مصدوميت‌هاي جزيي ثبت نشده و در نتيجه ابعاد مشكل در بخش سلامت كم­تر از واقع برآورد مي‌شود. نياز به سيستم‌ها پايش دقيق كه قادر به كشف اكثريت موارد مصدوميت‌هاي ترافيكي باشند، احساس مي‌شود.

Title: Epidemiologic survey of Extensive disasters in Islamic republic of Iran during recent decade (2000-2010)

http://knh.shmu.ac.ir/index.php/site/article/view/1010

Introduction: Natural disasters may strike a community at any time, causing death, injuries and material destruction. Knowledge of the epidemiology of disaster impacts is essential to determine effective responses. Iran is one of the most aired regions of the world, and suffers frequent natural disasters during the last decade were reported four major earthquake, one flood and the worst drought in the last third years. The aim of this study was the evaluation of the extensive disasters in Iran. Methods: For the collection of data has been used of the CRED-EMDAT international database and DesInventar. All of extensive natural disasters (death>50, affected>500) for deaths and number of affected people was assessed by province and city in ten recently years (2000-2010).  Results: The total deaths, affected people were 208 and 1827, respectively. About 50 disasters were happened in this period that 15 of them were extensive and the most of frequent were hydro metrological (53%) and 47%of them were me theological. All province of country has been reported one or more disasters. Conclusion: The results showed that the all kind of major disaster in Iran region has been reported in last decade. Conducting disaster risk reduction actions would be priority in Manager Organizations.

Title: General health state in patients with atypical chest pain

http://knh.shmu.ac.ir/index.php/site/article/view/1011

Introduction: It has been reported that atypical chest pain could have some relationship with panic disorders, anxiety and some other psychological problems. The present case control study is performed to evaluate general health state of patients with atypical chest pain and to study the most usual accompanying symptoms. Methods: Patients with less than 35 years old were included in the study. Patients with atypical chest pain were included as chest pain group if the probability of ischemic heart disease was low according to ACC/AHA guideline or they had negative stress tests. Control group were age and sex matched. General health questionnaire 28 (GHQ-28) were given to both groups and other data were gathered through interview. Results: Seventy patients with chest pain and 70 controls were included. Mean age of study group was 24.9 ± 6.4 years and 87.1% were female and 73.8% lived in the cities. There were no significant difference in history of diabetes mellitus, cigarette smoking, hyperlipidemia, drug consumption, family history, intake of coffee. Frequency of point chest pain was 33.8%, very short chest pain 58.8%, permanent chest pain 11.8%, chest tenderness 7.35%, pleuretic chest pain 17.65%, and radiation of chest pain to epigastr was 5.88%. Mean scores of GHQ-28 was 30.7 ± 14.5 in chest pain group and 23.6 ± 12.2 in control group (P=0.002). Palpitation and dyspnea were more prevalent in chest pain group (63.24% palpitation in chest pain group VS 24.64% in controls and 57.35% dyspnea in chest pain group VS 15.94% in controls), but desire for deep breath and difficulty in deep breath was not significantly different. Conclusion: The results of the present study confirms that patient with atypical chest pain have worse general health state. It is not clear that worse general health state is a reason of atypical chest pain or an incidental concomitant finding. Also the present study showed that palpitation and dyspnea may coexist with atypical chest pain. Whether these findings are due to more prevalent psychological problems in the patient with chest pain or not needs to be studied in future.

Title: بررسي همبستگي بين هوش هيجاني و کیفیت زندگی در دانشجویان دانشگاه علوم پزشکی قم در سال 1388

http://knh.shmu.ac.ir/index.php/site/article/view/1012

مقدمه: هدف اصلی بهداشت روانی پیشگیری از وقوع ناراحتی­ها و حفظ سلامت روانی است. هوش هیجانی به­عنوان یک موضوع جدید در حوزه روانشناسی مورد توجه قرار گرفته است. مطالعاتی که در این زمینه انجام شده است، مبین نقش هوش هیجانی و مؤلفه­های آن در جنبه­های مختلف زندگی فرد همچون پیشرفت تحصیلی، ازدواج، شغل و روابط اجتماعی می­باشد. امروزه این عقیده مطرح است فردی که دارای هوش هیجانی است بهتر می­تواند بر مشکلات زندگی غلبه کند به­طور مؤثرتری هیجانات خود را مهار کند که این هر دو سهم زیادی در تأمین بهداشت روانی خوب و کامل دارند، لذا این مطالعه به­منظور تعیین رابطه بین میزان هوشبهر هیجانی و کیفیت زندگی دانشجویان در دانشگاه علوم پزشکی قم انجام شد. مواد و روش­ها: این مطالعه، یک مطالعه توصیفی- تحلیلی از نوع مقطعی است. جامعه آماری شامل کلیه دانشجویان دانشگاه علوم پزشکی قم در سال تحصیلی 89-88 می­باشد. روش نمونه­گیری به­صورت تصادفی ساده از میان دانشجویان فوق انتخاب گردید. ابزار گردآوري داده­ها، پرسش­نامه مشخصات فردي، پرسش­نامه هوشبهر هيجاني بار- اُن و پرسش­نامه کیفیت زندگی بود. پس از جمع­آوری اطلاعات داده­ها با استفاده از ضریب همبستگی پیرسون و t توسط نرم­افزار SPSS مورد تجزیه و تحلیل قرار گرفت. يافته­ها: میانگین و انحراف معیار نمره کیفیت زندگی در نمونه فوق به­ترتیب 38/89 و 88/11 به­دست آمد 25% نمونه فوق نمره کیفیت زندگی بیش­تر از 95 داشتند در حالی­که 25% نیز نمره کیفیت زندگی کم­تر از 82 دارند. بین نمره کیفیت زندگی و هوش هیجانی ارتباط معناداری مشاهده شد (001/0>P). بین متغیرهای نمره کیفیت زندگی و جنس ارتباطی مشاهده نشد (790/0=P). بین متغیرهای نمره کیفیت زندگی و تأهل ارتباطی مشاهده نشد (637/0=P).    نتیجه­گیری: هوش هیجانی می­تواند سبب افزایش سلامتی، رفاه، ثروت، موفقیت، عشق و شادی گردد. افرادی با هوش هیجانی بالا توانایی اداره مطلوب خلق و خوی، وضع روانی، کنترل تکانه­ها، همدلی، آگاهی یافتن از احساسات خود و دیگران دارند و عاملی است که هنگام شکست ناشی از دست نیافتن به هدف در شخص امید و انگیزه ایجاد می­کند. تمام موارد ذکر شده نشانه سلامت فرد است که به­طور مستقیم و غیر مستقیم بر کیفیت زندگی اثر مثبت می­گذارند.

Title: بررسی ميزان افسردگی و عوامل مرتبط با آن در زنان مراجعه¬کننده به واحد بهداشت خانواده

http://knh.shmu.ac.ir/index.php/site/article/view/1013

مقدمه: افسردگی شايع­ترين اختلال روانپزشکی است و ناتوانی حاصل از آن بيش از ديگر بيماري­های مزمن می­باشد هم­چنين وقوع همزمان بالايی با ساير اختلالات جسمی و روانپزشکی دارد، از اين­رو مطالعه حاضر با هدف تعيين ميزان افسردگی و عوامل مرتبط با آن در زنان مراجعه­کننده به واحد بهداشت خانواده مراکز بهداشتي درماني شهر تهران طراحی و اجرا شد. مواد و روش­ها: اين پژوهش يک مطالعه توصيفی از نوع مقطعی بود که تعداد 811 نفر از زنان مراجعه­کننده به ­روش نمونه­گيری طبقه­ای تصادفی انتخاب شدند. ابزار گردآوری اطلاعات پرسش­نامه بود که از طريق مصاحبه تکميل گرديد، برای تجزيه و تحليل اطلاعات از آمار توصيفی و استنباطی شامل آزمون مجذور کای و آزمون دقيق فيشر استفاده شد. يافته­ها: ميزان افسردگی 1/37% بود (افسردگی خفيف 7/28%، افسردگی متوسط 2/8% و افسردگی شديد 2/0%). بين افسردگی و برخی از عوامل شامل شغل (006/0=P)، تحصيلات (001/0>(P، تحصيلات همسر (001/0=P) و وضعيت اقتصادی (001/0>(P ارتباط معنی­دار وجود داشت. نتيجه­گيری: با توجه به شيوع بالاي افسردگی در اين پژوهش و وجود ارتباط بين برخی از عوامل با افسردگی و از آنجايی که مداخله برای کاهش افسردگی از نتايج نامطلوب آن بر سلامت زنان می­کاهد، غربالگری افسردگی به خصوص در زنان در معرض خطر (با توجه به مشخصات دموگرافيک آن­ها) توصيه می­شود.

Title: بررسي وضعيت مصرف سيگار و عوامل مؤثر بر گرايش به ترک در دانشجويان خوابگاه¬هاي دانشگاه علوم پزشکي تبريز

http://knh.shmu.ac.ir/index.php/site/article/view/1014

مقدمه: ترک سيگار در جوانان و نوجوانان يکي از اولويت­هاي برنامه­هاي پيشگيري مي­باشد. هدف مطالعه حاضر تعيين شيوع مصرف سيگار، بررسي تجربيات ترک سيگار و عوامل مؤثر بر گرايش به ترك سيگار در بين نمونه­اي از دانشجويان دانشگاه علوم پزشکي مي­باشد. مواد و روش­ها: در مجموع 523 نفر (293 زن، 246 مرد) از دانشجويان در يک مطالعه مقطعي شرکت کردند. ابزار جمع­آوري اطلاعات يک پرسش­نامه خود ايفا بود که شامل سؤالاتي در مورد اطلاعات دموگرافيکي سؤالاتي به­صورت باز و بسته جهت تعيين وضعيت مصرف سيگار، تجربيات ترک، گرايش به ترک، مصرف الکل و مصرف مواد اعتياد آور قبل و بعد از شروع مصرف سيگار بود. داده­ها توسط آزمون­هاي t-test و و دقيق فيشر با استفاده از نرم­افزار SPSS آناليز شدند. يافته­ها: شيوع کلي مصرف سيگار9/8% (18 % مردان و 4/1% زنان) بود. بين جنس و وضعيت مصرف سيگار و هم­چنين بين بين مصرف الکل و مصرف سيگار رابطه آماري معناداري وجود داشت (001/0P<). مهم­ترين دلايل شروع مصرف سيگار، کنجکاوي، کسب تجربه جديد و امتحان کردن (76/37%)، کسب لذت و سرگرمي (48/17%)، مشکلات روحي و رواني و آرامبخشي (08/16%)، دوستان سيگاري ( 38/15%)، ناآگاهي و بي­تجربگي (89/4%) بودند. 16/54% مصرف­کنندگان کنوني سابقه اقدام به ترک داشتند. آگاهي از مضرات سيگار (47/43%)، عدم وابستگي و علاقه به سيگار (73/21%)، تنفر از دود سيگار (04/13%)، توصيه­هاي ديگران (69/8%)، موقعيت اجتمايي (69/8%) مهم­ترين دلايل ترک سيگار بودند. مهم­ترين دلايل عدم موفقيت در ترک مشکلات مادي و اجتماعي (26%)، افسردگي و مشکلات عصبي (23%)، وابستگي روحي و رواني (23/19%)، و دوستان سيگاري (38/15%) بودند. از عوامل تأثيرگذار در ايجاد انگيزه براي ترک، ضرر جسماني ناشي از مصرف سيگار، اعتقاد به مضر بودن سيگار، بيماري­هاي مرتبط با سيگار و موقعيت اجتماعي خود به ترتيب بيش­ترين تأثير را داشتند. شيوع مصرف اکثر مواد اعتيادآور بعد از شروع مصرف سيگار در مقايسه با قبل از آن بالا بود. نتيجه­گيري: با شناخت هرچه بيش­تر عوامل مؤثر در ايجاد انگيزه براي ترک سيگار مي­توان برنامه­هاي پيشگيري بسيار کارآمدتري را براي جوانان طراحي کرد.

Title: هزينه اثربخشي راه¬کارهاي پيشگيري اوليه از ديابت نوع دوم

http://knh.shmu.ac.ir/index.php/site/article/view/1015

مقدمه: ديابت نوع دوم يکي از عمده­ترين مسايل بهداشتي درماني کشورها است که هزينه درماني و از کارافتادگي بسيار بالايي دارد. هم­چنين از لحاظ هزينه­هاي اقتصادي بايد گفت در تمام سيستم­هاي مراقبت بهداشتي هزينه يک فرد ديابتي 2 تا 4 برابر بيش­تر از هزينه يک فرد عادي است. 15 تا 25 درصد کل هزينه­هاي سلامت در کشورهاي پيشرفته صرف درمان، پيشگيري و کنترل ديابت شده و 16 درصد هزينه­هاي بيمارستاني به ديابت تعلق دارد. هدف از مقاله حاضر معرفي راه­کارهاي پيشگيري اوليه و اثر بخشي هر کدام از اين راه­کارها است. مواد و روش­ها: مقاله گردآوري شده حاصل بازنگري سيستماتيک مقالات، گزارش­هاي سازمان جهاني بهداشت و ساير کشورها در ارتباط با راه­کارهاي پيشگيري اوليه ديابت نوع دو با تأکيد بر هزينه اثربخشي آن­ها است. يافته­ها: شش برنامه گسترده پيشگيري اوليه ديابت نوع دو مورد بحث است، که نتايج براساس افزايش سن بروز ديابت و افزايش عمر (سال­هاي زندگي) افراد ديابتي تخمين­زده شده است. برنامه شماره 1: اصلاح سبک زندگي کاري به­صورت برنامه گروهي، برنامه شماره 2: برنامه رسانه­اي سبک زندگي با حمايت اجتماعي، برنامه شماره 3: استفاده از روش­هاي جراحي براي افراد مبتلا به چاقي مرضي، برنامه شماره 4: برنامه­هاي اصلاح رفتاري براي افراد مبتلا به چاقي مزمن، برنامه شماره 5: برنامه براي زنان مبتلا به ديابت حاملگي، برنامه شماره 6: توصيه­هاي اصلاح سبک زندگي براي افراد مبتلا به ريسک فاکتورهاي ديابت نوع دو. نتيجه­گيري: در مورد تعداد سال­هاي پيشگيري از ديابت، استفاده از برنامه شماره 3 بهترين اثربخشي و برنامه شماره 6 کم­ترين اثربخشي را داشته است. هم­چنين برنامه شماره 3 بيش­ترين اثربخشي و برنامه شماره 6 کم­ترين اثربخشي را در زمينه افزايش طول عمر افراد ديابتي داشته­اند. از نظر هزينه پيشگيري از ديابت به­طور ساليانه، برنامه شماره 3 با توجه به اينکه گروه هدف کوچک مي­باشد بسيار زياد است. اما برنامه شماره 1 با صرفه­ترين هزينه پيشگيري را داشته است. در زمينه هزينه هر سال زندگي حفظ شده، برنامه شماره 4 بيش­ترين هزينه اثربخشي را داشته­است. به­طور کلي خالص هزينه اثربخشي برنامه­هاي شماره 1، 2 و 4 بهترين اثر بخشي را داشته­اند. بنابراين جهت پيشگيري از ديابت نوع دو با توجه به محدود بودن منابع بخش بهداشت در کشور­هاي در حال توسعه مي­توان از اصلاح سبک زندگي افراد در محيط کار، بهره­گيري از برنامه­هاي رسانه­اي و حمايت­هاي اجتماعي و اصلاح رفتار مي­توان استفاده نمود.

Title: عدالت در سلامت عمومی (مصرف بهینه در سلامت)

http://knh.shmu.ac.ir/index.php/site/article/view/1016

مقدمه: این مقاله نتایج بررسی مقطعی را در تعیین هزینه­های بیماران دریافت کننده­ی خدمات بهداشتی درمانی در مؤسسات خصوصی و دولتی، تجزیه و تحلیل کرده است. مصرف بهنه در بهداشت به درست مصرف کردن کالا­ها و خدمات بهداشتی درمانی و هزینه­های جیبی کاربران اشاره دارد. ارتباط هزینه­های مستقیم و غیرمستقیم در صنعت سلامت بین عدالت و مصرف بهینه در این بخش به­صورت سیستماتیک باز نگری شده است. مدل­های مصرف بهینه و ارايه راه حل برای کاهش در هزینه­های شخصی مستلزم تحلیل تعامل، بین عدالت و دسترسی با هزینه­های بهداشتی درمانی است. مواد و روش­ها: این مقاله در کشور­های با درآمد متوسط و فقیر تطبیق داده شده است و با مطالعه سیستماتیک آنالیز شده است. متغیرهای وابسته، هزینه­های جیبی(کاربران پرداخت­کننده) بودند و متغیرهای مستقل هم، بیمه، نوع شرکت بیمه­ای و درآمد آن­ها بوده است. يافته­ها: یافته­های تجربی نشان می­دهد که نیاز به تحلیل جزيی­تری در هزینه­های کاربران خدمات بهداشتی درمانی به­خصوص در کشورهای با درآمد متوسط و کشورهای فقیر وجود دارد که این موضوع هم به ارتباط بخش خصوصی و دولتی بستگی دارد. با آنالیز مقاله می­فهمیم که هزینه­های جیبی با بی عدالتی مصرف شده که این در نهایت باعث کاهش درآمد ملی و در نتیجه کاهش توسعه اقتصادی خواهد شد. هدر رفتن سوبسید­های دولت به علت عدم نظارت باعث عقب­ماندگی خواهد شد. نتیجه­گیری: پزشکان (سرمایه­های انسانی) نقش مهمی را در این هزینه­ها ایفا می کند به­طوری که با ترافیک ویزیت و نسخه سعی درکسب درآمد بیش­تر دارند، پس با این وجود تحلیل و بازنگری در هزینه­ها لازم است. همه­ی این مطالب نقش و اهمیت عدالت در سلامت را مدنظر قرار می­دهد. وجود عدالت به خصوص در صنعت بهداشت و درمان از طریق دسترسی به خدمات بهداشتی باعث کاهش هزینه­ها در سیستم سلامت شده و به پیشرفت کشور کمک خواهد کرد، از آنجا که تعامل پیچیده و اساسی بین سلامت و توسعه وجود دارد،وجود ارتباط بین تمامی بخش­های کشور لازم و ضروری است.. نقش سیاست کلان اقتصادی در بهداشت و درمان و وجود برنامه­ریزی تشریفاتی در نابرابری خدمت سلامت حائز اهمیت است.

Title: بررسی تأثیر کمک¬های حمایتی تغذیه¬ای کمیته امداد امام (ره) روی اصلاح اختلال رشد کودکان 6-1 سال در شهر نکا

http://knh.shmu.ac.ir/index.php/site/article/view/1017

مقدمه: سوءتغذیه در کودکان و به­ویژه اطفال زیر6 سال، یکی از مهم­ترین دلایل اختلالات رشد در آن­ها می­باشد. بررسی­های انجام گرفته در مناطق مختلف کشور ما دلالت بر وفور نسبتاً گسترده سوءت غذیه در اطفال زیر 6 سال دارد، لذا سوء تغذیه در اطفال زیر 6 سال یکی از مسایل بهداشتی درخور توجه در کشور ماست. به­طور کلی مسأله تغذیه و به همین صورت سوء تغذیه یک موضوع چند وجهی است که عوامل زیادی مانند بهداشت، فقر، دسترسی نداشتن به غذا، ناآگاهی، عادت­های غلط غذایی، بروز بیماری و مراقبت ناکافی، محیط آلوده و ناسالم می­توانند در آن دخالت مستقیم یا غیر مستقیم داشته باشند. با توجه به اتیولوژی مسايل تغذیه­ای، فائق آمدن بر آن عمدتاً مستلزم تنظیم برنامه­های فرابخشی می­باشد، که در آن بخش­های مربوطه بتوانند نقش­های مؤثر را ایفا نمایند. در این مقاله سعی شده است تا نتایج بررسی در رابطه با تأثیر برنامه مشترک اجرا شده کمیته امداد امام (ره) و وزارت بهداشت، درمان و آموزش پزشکی در شناسایی و حمایت تغذیه­ای از کودکان دارای اختلال رشد در شهر نکا از استان مازندران گزارش گردد. مواد و روش­ها: مطالعه حاضر از نوع کوهورت تاریخی بوده است. لذا روند تغییر رشد کودکان زیر 6 سال دارای سوءتغذیه در شهر نکا که تحت پوشش برنامه مورد مطالعه بوده­اند، با کودکان مشابه در شهرساری به­عنوان شهر همجوار، که فاقد مداخله مشابهی بوده است، مورد مقایسه قرار گرفته­اند. ابزار گردآوری اطلاعات، چک لیستی بوده است که بر مبنای اطلاعات موجود در پرونده خانوار طراحی و به جمع­آوری اطلاعات مبادرت شده است. در این مطالعه 140 پرونده مربوط به خانوارهای دارای کودک 6- 1 سال مبتلا به اختلال رشد در شهر نکا، به­علاوه همین تعداد پرونده کودک دچار اختلال رشد در شهرساری که شرایط اجتماعی- اقتصادی تقریباً مشابهی داشته­اند (جمعاً پرونده خانوار 280 کودک 6- 1 ساله درارای اختلال رشد)، مورد بررسی و مقایسه قرار گرفته­اند. يافته­ها: این مطالعه نشان داد که اختلاف روند رشد کودکان بین دو ناحیه شهری نکا و ساری معنادار نمی­باشد، ولی به­طور کلی روند رشد کودکان رو به بهبودی بوده است و در شهر نکا وضعیت بهتری از شهر ساری مشاهده شده است. نتیجه­گیری: مداخله تأثیر معنادار در روند رشد کودکان نداشته است ولی دارای تأثیر بیش­تر نسبت به شهر مشابه بوده است، بررسی دقت و شدت مداخله از نظر تأثیر بر روند اصلاح رشد کودکان در مطالعات بعدی پیشنهاد می­شود.

Title: استاندارسازی و اعتبارسنجی پرسش¬نامه کیفیت زندگی و ارزیابی آن در بیماران دیابتی نوع 2

http://knh.shmu.ac.ir/index.php/site/article/view/1018

مقدمه: دیابت می­تواند بر عملکرد فیزیکی، توسعه عوارض بیماری، وضعیت روانی، ارتباطات فردی، خانوادگی و اجتماعی، عملکرد جنسی و درک از سلامتی تأثیرگذار باشد. با توجه به افزایش روزافزون بروز و شیوع دیابت نوع 2، و اهمیتی که تأثیر این بیماری بر روی تمام ابعاد زندگی افراد مبتلا دارد این مطالعه با هدف ساخت پرسش­نامه استاندارد اندازه­گیری کیفیت زندگی در بیماران دیابتی نوع 2 انجام گردید. مواد و روش­ها: این پژوهش یک مطالعه توصیفی- تحلیلی از نوع مقطعی است.حجم نمونه مورد بررسی به تعداد 254 نفر بوده که به شیوه تصادفی دو مرحله­ای انتخاب شدند. برای ارزیابی روایی و پایایی پرسش­نامه از اعتبار محتوی، تحلیل عاملی و ضریب آلفای کرونباخ و برای تجزیه و تحلیل داده­ها از آمار توصیفی و آزمون­های تی مستقل، کای اسکور و آنالیز واریانس و رگرسیون خطی چندگانه و سطح اطمینان 95% در نرم­افزار SPSS استفاده گردید. یافته­ها: ضریب همیستگی برای سنجش اعتبار محتوای پرسش­نامه، با استفاده از روش­هامیلتون، 100% به­دست آمد. پایایی پرسش­نامه با استفاده از ضریب آلفای کرونباخ 922/0 (935/0 – 907/0):CI 95% به­دست آمد. در تحلیل عاملی انجام شده مشخص گردید که چهار فاکتور اصلی وجود داشته و بر حیطه­های مورد نظر منطبق و متناسب می­باشند در مجموع 8/89 % واریانس متغیرهای مورد بررسی را توصیف می کنند. نتیجه­گیری: پرسش­نامه مورد استفاده از روایی و پایایی مطلوبی برخوردار بوده و توانسته است کیفیت زندگی را در بیماران دیابتی نوع 2 اندازه­گیری و گزارش نماید.

Title: محاسبه سرانه كاركرد كاركنان شاغل در مراكز بهداشتي درماني و خانه¬هاي بهداشت شهرستان شاهرود

http://knh.shmu.ac.ir/index.php/site/article/view/1019

مقدمه: اولين قدم در ايجاد هر نوع كنترل و ارزيابي عملكرد ايجاد استاندارد براي اندازه­گيري نحوه انجام كار است. در محاسبه هزينه تمام شده خدمات سلامت، استاندارد نمودن خدمات و فعاليت­ها و زمان، امري زيربنايي است. مطالعه به­منظور تعيين سرانه كاركرد كاركنان شاغل در مراكز بهداشتي درماني و خانه­هاي بهداشت شهرستان شاهرود در سال 1387 انجام شد. مواد و روش­ها: اين مطالعه از نوع پژوهش­هاي کاربردی است که به شیوه توصيفي- مقطعي (cross-sectional) انجام شد. در اين مطالعه جهت دقت بیش­تر کار کلیه مراکز بهداشتی درمانی شهری و روستایی (28 مرکز) و تمامی خانه­های بهداشت (63 واحد) را به روش سرشماری مورد پژوهش قرارگرفت. استاندارد نمودن فعالیت­ها و تعیین زمان مورد نیاز جهت انجام فعالیت با استفاده از نظرات مجریان، کاردان­ها و کارشناسان سطوح بالاتر و متخصصین به روش PERT صورت گرفت. اطلاعات مورد نياز در قالب فرم­هاي جمع­آوري داده­ها گردآوري و وارد نرم­افزار Excelشد. جهت تحليل داده­ها از نرم­افزار Excel استفاده گردید. نمايش داده­ها به­وسيله جداول توزيع فراواني و نمودار صورت گرفت. يافته­ها: یافته­های حاصل از تحلیل داده­ها نشان داد که (43/44%) 259 نفر ار افراد مورد مطالعه را مردان و (57/55%) 324 نفر را زنان تشکیل می­دادند. تعداد 133 نفر (81/22%) از نیروها در خانه­های بهداشت، تعداد 149 نفر (56/25%) در مراکز بهداشتی درمانی روستایی، تعداد 76 نفر (04/13%) در مراکز بهداشتی درمانی شهری، تعداد 120 نفر (58/20%) در مراکز بهداشتی درمانی شبانه­روزی، تعداد 15 نفر (57/2%) در پایگاه­های بهداشتی و 90 نفر (44/15%) در ستاد معاونت بهداشتی شهرستان شاغل بودند. سرانه ساعت کار مفید پرسنل (با نگرش انجام کار مطابق استاندارد) در خانه بهداشت 4 ساعت و سی دقیقه و 6 ثانیه و در مرکز بهداشتی درمانی روستایی 3 ساعت و 34 دقیقه و 8 ثانیه، در مرکز بهداشتی درمانی شهری برابر با 3 ساعت و 23 دقیقه و 40 ثانیه، در مرکز بهداشتی درمانی شبانه روزی برابر با 5 ساعت و 43 دقیقه و 20 ثانیه و در پایگاه بهداشتی برابر با 2 ساعت و 48 دقیقه و در مجموع شهرستان برابر با 3 ساعت و 45 دقیقه و 6 ثانیه بود. نيروهاي موجود به ازاي هر دقيقه كار موظف 459 ريال و هر دقيقه اشتغال به­كار (كار مفيد) 854 ريال حقوق و دستمزد دريافت نموده بودند. نتيجه­گيري: نتایج حاصل از پژوهش حاکی از آن است که از نیمی از توانایی کارکنان استفاده شده و غنی­سازی شغلی و شفاف­سازی فرآیندهای کار می­تواند نقش مهمی در افزایش بهره­وری داشته باشد.

Title: کاربرد فاکتور بیز در پزشکی مبتنی بر شواهد

http://knh.shmu.ac.ir/index.php/site/article/view/1020

فرض کنید  احتمال پیشین برای مدل  باشد. برای هر دو مدل دلخواه مثل  و  فاکتور بیز به­صورت زیر تعریف می­شود:   فاکتور بیز مقیاسی بسیار قابل درک با کاربرد وسیع در بسیاری از زمینه­های علمی است. در واقع فاکتور بیز شواهدی را به نفع یک واقعه خاص فراهم می آورد. به­عنوان مثال اگر  باشد، می­توان به این نتیجه رسید که بخت حضور مدل اول دو برابر مدل دوم است. آنالیزهای غیر بیزی بر اساس مدلی است که در ابتدا انتخاب و سپس تحلیل­های بعدی، بر اساس همان مدل اولیه انجام می­شود. اما این واقعیت غیر قابل چشم پوشی است که تحت این مدل­ها احتمال کم برآوردی به وجود می­آید. از سویی شاید انتخاب مدل با سوگیری انجام شود و ناگزیر برآوردهای انجام شده نیز با اریبی و پراکندگی­ها بسیار خوش­بینانه بیان شوند. این در حالی است که استفاده از استنباط بیزی (حتی زمانی که توزیع پیشین بسیار هموار در نظر گرفته می­شود) برآوردهایی را ارايه می­دهند، که به واقعیت نزدیک­تر هستند. از آنجا که در علوم طبیعی و مخصوصاً پزشکی اهمیت بسیار زیادی به اطلاعات قبلی می­دهند، تئوری بیز و فاکتور بیز می­توانند در زمره پرکاربردترین استنباط­های آماری قرار گیرند. به­عنوان مثال زمانی که پزشک می­خواهد احتمال ابتلا به یک بیماری را محاسبه کند، یا زمانی که بین دو بیماری برای مریض خود در شک است، با استفاده از اطلاعات گذشته بیمار، میزان شیوع بیماری، و اطلاعات جانبی مفید به محاسبه فاکتور بیز پرداخته و شواهد کافی را برای نسبت دادن هر بیماری جمع­آوری می­کند و با احتمال بالاتری تشخیص خود را بیان می­کند. آنچه در این مقاله سعی داریم بدان اشاره کنیم استفاده از فاکتور بیز در پزشکی مبتنی بر شواهد است.

Title: طرح¬های مطالعاتی در اپیدمیولوژی آلودگی هوا

http://knh.shmu.ac.ir/index.php/site/article/view/1021

مقدمه: پدیده مه انگلستان نقش آلودگی هوا را در افزایش مرگ­ومیر نمایان ساخت. ولی در دهه 70 و 80 مطالعات متعدد نتوانستند ارتباط مناسبی میان آلودگی هوا و مرگ­ومیر مرتبط با آن برقرار سازند. از آنجا که در سال­های اخیر روش­های مطالعاتی متعددی جهت بررسی ارتباط میان آلودگی هوا و پیامدهای بهداشتی مورد استفاده قرار گرفته است این مقاله به مرور و مقایسه انواع مطالعات آلودگی هوا می­پردازد. مواد و روش­ها: این مطالعه مروری به بررسی ویژگی­ها، نقاط قوت و ضعف انواع طرح­های مورد استفاده در مطالعات آلودگی هوا می­پردازد. یافته­ها: مطالعات آلودگی هوا شامل مطالعات حیوانی و مطالعات انسانی هستند. مطالعات انسانی شامل مطالعات تجربی (مطالعات خانه‌ای) و مطالعات اپیدمیولوژیک (مشاهده‌ای) بوده كه در بين مطالعات اپیدمیولوژیک، مطالعات پانل، مورد- متقاطع، مقطعی و سری زماني برای برآورد اثرات حاد آلودگی هوا مناسب هستند در حالیکه مطالعات همگروهی قادر به برآورد اثرات حاد و هم­چنين اثرات مزمن هستند. مطالعه سری زمانی برای کشف اثرات مخرب آلودگی هوا در سطوح پایین‌تر از سطح استاندارد، مطالعات مقطعی برای کشف اثرات حاد پیامدهای بهداشتی مرتبط با مواجهه و نیز برای مواجهات ثابت در طول زمان، مطالعه مورد- متقاطع برای شناسایی گروه‌های مستعد و حساس به آلودگی هوا، مطالعه پانل برای مطالعه اثرات بهداشتی کوتاه مدت و در برخی موارد نادر و طولانی مدت آلاینده‌ها بخصوص در زیرگروه‌‌های حساس و مستعد و مطالعات کوهورت جهت ارزیابی اثرات تجمعی آلودگی هوا در طول سالیان متمادی مناسب است. نتیجه­گیری: با توجه به معایب و مزایای هر طرح، انتخاب بهترین طرح بستگی به سوال مورد پژوهش و نوع داده‌های در دسترس دارد. مسلماً هیچ طرح مطالعاتی در تمام موارد بهينه نبوده و هر طرحی برای نوع خاصی از اثر پیامد و مواجهه مناسب‌تر است.

Title: ساخت مدل پيشگويي¬كننده ميرايي در بيماران مبتلا به HIV/AIDS مركز مشاوره بيماري¬هاي رفتاري دانشگاه علوم پزشكي شيراز با كنترل داده¬هاي مفقود شده آن¬ها در طي سال¬هاي 1383 -1388

http://knh.shmu.ac.ir/index.php/site/article/view/1022

مقدمه: يكي از مهم­ترين چالش­هاي مراقبت و درمان بيماران مبتلا به HIV/AIDSتشخيص به هنگام عوامل پيشگويي كننده مرگ­ومير مي­باشد. مدل رگرسيوني شيوه مناسبي براي ارزيابي عوامل خطر مرتبط با بيماري است. اگرچه وجود داده­هاي مفقود شده ساختن مدل را دشوار مي­كند. هدف اين مقاله ايجاد مدل­هاي تشخيصي براي پيش­بيني مرگ­ومير در بيماران HIV/AIDS است. مدل را پس از حذف داده­هاي مفقود شده ايجاد كرده (C-C model)و سپس از شيوه استنادسازي (MICE technique) استفاده كرديم. مواد و روش­ها: اين مطالعه بر روي 1460 شخص آلوده بهHIV كه در طي سال­هاي 1383 الي 1388 به مركز مشاوره بيماري­هاي رفتاري دانشگاه علوم پزشكي شيراز مراجعه كرده بودند انجام شد. از 15 متغير كه به­عنوان عامل خطر مطرح بودند، تنها متغير­هايي كه در آناليز تك متغيره داراي P-value كم­تر از 25 % بودند براي مدل آناليز چندگانه انتخاب شدند. در مدل C-Cبيماراني كه حتي داراي يك مشاهده مفقود شده بوده را حذف كرديم. در مرحله بعدي از مدل MICE استفاده و هر مقدار مفقود شده را به­وسيله 10 مقدار استناد سازي شده جايگزين كرده و چندين داده مرجع استناد شده را توليد كرديم.درهر دو مدل از شيوه حذف روبه عقب استفاده شد. يافته­ها: در آناليز با شيوه C-C حجم نمونه به 475 كاهش يافته و تنها P.V معناداري را براي سه متغيير پيش­گويي­كننده (با و بدون علامت ايدز، متادون و تاريخچه زندان) يافتيم. در مدل MICE 5 متغير ديگر نيز معنادار شدند. نتيجه­گيري: داده­هاي مفقود شده مشكل بسياري از اطلاعات مراكز بهداشتي و درماني است. در آناليز با مدل C-C ما داده­هاي مفقود شده راناديده گرفتيم كه اين مسأله منجر به از دست رفتن توان آماري براي برخي متغيرها شده، در حالي­كه استناد سازي با شيوه­هايي مانند MICE برخي از متغيرهاي ديگررا نيز به سطح معناداري رساند. ما به نتيجه رسيديم كه مدل MICE تكنيكي بسيار قوي بوده و آن را براي داده­هاي مفقود شده توصيه مي­نماييم.

Title: ارزیابی دقت سنی و جنسی داده¬های جمعیتی زیج حیاتی و سرشماری ایران

http://knh.shmu.ac.ir/index.php/site/article/view/1023

مقدمه: داده­های جمعیتی یکی از مهم­ترین داده­های لازم برای فرایند برنامه­ریزی برای ارایه خدمات مختلف سلامت به جامعه می­باشند. در ایران به­خصوص در مناطق روستایی، زیج حیاتی برای جمع­آوری و نشان دادن وقایع حیاتی طراحی شده است. در این سیستم، داده­های پایه جمعیتی به­وسیله بهورزان جمع­آوری و در زیج حیاتی وارد می­شوند. هدف این مطالعه ارزیابی میزان دقت سنی و جنسی داده­های جمعیتی گزارش شده در زیج حیاتی و مقایسه آن با داده­های سرشماری انجام شده در سال 1385 با استفاده از شاخص سازمان ملل می­باشد. مواد و روش­ها: در این مطالعه برای ارزیابی کیفیت داده­های جمعیتی از شاخص سازمان ملل (UN Index) که شامل محاسبه نسبت جنسی و نسبت جنسی برای گروه­های 5 ساله تا سن 70 + سالگی است، استفاده شده است. معیار این شاخص برای ارزیابی عبارت است از: کم­تر از 20 دقیق، بین 20 تا 40 غیر دقیق و بیش­تر از 40 بسیار غیر دقیق. یافته­ها: نتایج مطالعه نشان داد که شاخص سازمان ملل برای زیج حیاتی عبارت بود از 28.63 و برای سرشماری عبارت بود از 30.34 که به این معنی است که داده­های جمعیت حاصل از زیج حیاتی و سرشماری از دقت لازم برخوردار نیستند. این شاخص برای زیح حیاتی روستایی استان­های مازندران (84/28) تا کهگیلویه و بویر احمد (33/83) متغیر بوده است. حدو د 70 درصد از استان­ها شاخص سنی و حنسی آن­ها د قت لازم را نداشته­اند. به­خصوص داده­های استان های کهگیلویه و بویراحمد،اردبیل، مرکزی، ایلام، آذربایجان غربی و هرمزگان (بین 60 تا -84). محاسبه شاخص سازمان ملل برای مناطق روستایی بر اساس داده­های سرشماری سال 1385 نشان داد که نقش ثبات جمعیت مؤثر است. به­طور مثال شاخص سازمان ملل برای استان­های کهگیلویه و بویراحمد، اردبیل، مرکزی، آذربایجان غربی و هرمزگان برا اساس داده­های سرشماری به­ترتیب 64/59، 57/33، 66/28، 55/25 و 55/49 می­باشد. نتیجه­گیری: اگر چه شاخص سازمان ملل ممکن است محدودیت­هایی داشته باشد، ولی معیار مفیدی برای بررسی دقت داده­های جمعیتی از نظر ساختار سنی و جنسی می­باشد و نتایج این مطالعه نشان داد که تلاش­های بعدی برای ارتقای داده­های جمعیتی در مناطق روستایی نیاز است.

Title: اپیدمیولوژی سرطان¬ها و مرگ¬ومیر ناشی از آن¬ها بر اساس مطالعه ثبت سرطان بیمارستانی استان هرمزگان، 86-1381

http://knh.shmu.ac.ir/index.php/site/article/view/1024

مقدمه: آنچه امروز توجه به ثبت دقیق و اجرای روش­های پیشگیری و درمان سرطان را الزامی می­سازد تغییر الگوی بیماری­ها است. سرطان به­طور فزاینده­ای عاملی مهم در بار جهانی بیماری در دهه­های آینده خواهد بود. انتظار می­رود تعداد موارد جدید سرطان از 10 میلیون نفر در سال 2000 به 15 میلیون نفر در سال 2020 افزایش یابد که حدود 60% این موارد جدید در کشورهای کم­تر توسعه یافته خواهد بود. در کشورهای توسعه یافته سرطان دومین عامل شایع مرگ­ومیر است و شواهد اپیدمیولوژیک اشاره به ظهور روند مشابهی در کشورهای در حال توسعه دارد. در حال حاضر سرطان عامل 12% کل مرگ­ها در سرتاسر جهان است و در ایران، سومین عامل مرگ­ومیر می­باشد. بنابراین شناسایی اپیدمیولوژی سرطان­ها در مناطق مختلف کشور با توجه به موقعیت متفاوت جغرافیایی هر منطقه جهت تعین بار بیماری و میزان مرگ­ومیر ناشی از آن­ها ضروری به نظر می­رسد که در این مطالعه به اپیدمیولوژی سرطان­ها و مرگ­ومیر ناشی از آن­ها بر اساس مطالعه ثبت سرطان بیمارستانی استان هرمزگان پرداخته می­شود. مواد و روش­ها: این مطالعه از نوع توصیفی مبتنی بر داده­های حاصل از ثبت سرطان بیمارستانی در سال­های 86-1381 در بیمارستان­های آموزشی بوده است. جهت گردآوری داده­ها از نرم­افزار آماری تخصصی ویژه بخش مدارک پزشکی (ADS9) بیمارستان­ها استفاده گردید. داده­ها با استفاده از نرم­افزار SPSS تحلیل گردید. يافته­ها: تعداد کل نئوپلاسم­ها 1941 مورد بوده که در زنان 989 مورد (95/50%) و مردان 952 مورد (05/49%) بود و یک پیک در سال 1384 (537 مورد) دیده شد. تعداد مرگ ناشی از نئوپلاسم­ها 133 مورد بوده که 75 مورد (4/56%) مربوط به مردان و 58 مورد (6/43%) مربوط به زنان بوده است. بیش­ترین مرگ هم در سال 1384 (33 مورد) اتفاق افتاده است. نتیجه­گیری: بر اساس آمار کشوری سرطان­ها، از کل میزان بروز سرطان در 1383، حدود 8/0% مربوط به زنان و 8/0% مربوط به مردان مورد مطالعه می­باشد. الگوی منظمی از نظر میزان بروز بیماری و مرگ­ومیر در سال­های مورد مطالعه مشاهده نشد. با توجه به اینکه این مطالعه به­صورت ثبت سرطان مبتنی بر بیمارستان بوده بر اساس اطلاعات و بررسي­های انجام شده تخمين­زده مي­شود كه ميزان سرطان در استان بیش­تر از این مقدار باشد که باید مراکز ثبت سرطان در این استان فعال­تر عمل نمایند.

Title: بررسی اپیدمیولوژیک خشونت و ارتباط آن با سن و جنس و محل درگیری، استان اصفهان، سال 1387

http://knh.shmu.ac.ir/index.php/site/article/view/1025

مقدمه: خشونت، پرخاش و پرخاشگری را از این منظر که در محدوده روابط متقابل اجتماعی تجلی و بروز می­یابند می­توان پدیده­های اجتماعی- روانی تلقی نمود. خشونت به دنبال منازعه آسیبی است که به­طور عمدی توسط فرد یا گروه کوچکی از افراد اعمال می­گردد و یک مشکل جدی سلامت عمومی در بسیاری از کشورها است. طبق تخمین سازمان جهانی بهداشت در سال 2000 خشونت مسوول 1658000 مورد مرگ و بیش از 460 میلیون سال عمر تلف شده به علت مرگ وناتوانی (DALY) است. الگوی خشونت به دنبال منازعه و متغیرهای مرتبط با آن در کشورهای مختلف متفاوت بوده و تابع عوامل اجتماعی- اقتصادی، قانونی در دسترسی به سلاح می­باشد. موا د و روش­ها: این مطالعه توصیفی- تحلیلی گذشته نگر است. آمار و اطلاعات از طریق برنامه نرم­افزاری حوادث مرکز بهداشت استان اصفهان جمع­آوری و سپس تجزیه و تحلیل گردیده است. یافته­ها: آمار نشان می­دهد که در سال 1387 در کل 4569 موردخشونت اتفاق افتاده است که 4% کل حوادث را تشکیل می­دهد. از این میان 4108 مورد از خشونت اتفاق افتاده (90%) از سوی مردان و 461 مورد (10%) از سوی زنان اتفاق افتاده است. از نظر گروه­های سنی درگیر، گروه سنی 24-20 سال با مجموع 1307 مورد (30%) در رده اول و گروه سنی 19-15 سال با 900 مورد (7/19%) در رده دوم و گروه سنی 29-25 سال با 867 مورد (19%) و سپس گروه سنی 34-30 سال با 460 مورد (10%) بوده است و سایر گروه­های سنی در رده­های بعدی قرار دارند. از نظر محل درگیری بیش­ترین محل درگیری کوچه و خیابان با 2719 مورد (59%) سپس منزل با 711 مورد (5/15%) و پس از آن اماکن عمومی با 518 مورد (3/11%) و محل کار با 95 مورد (3/4%) بوده است و سایر موارد در صد کمی را شامل می­شود. از نظر منطقه حادثه 4095 مورد از خشونت­ها (89%) در مناطق شهری و 363 مورد (8%) در مناطق روستایی و 3% باقیمانده در مناطق شهری روستایی اتفاق افتاده است. نتیجه­گیری: مهم­ترین نکته درمطالعه این آمار به سن ایجاد خشونت بر می­گردد. بیش­ترین سن گرفتار در گروه جوان و فعال جامعه است که در صورت ایجاد آسیب­های جسمی جدی،علاوه بر هزینه­های درمان سبب وارد شدن صدمات اقتصادی به علت از کار افتادن نیروی فعال کار در جامعه می­گردند و نکته دیگر درگیری در جنس مذکر است. با توجه به اینکه بش­ترین موارد خشونت در مناطق شهری اتفاق افتاده است و این مسأله که خشونت را ناشی از عوامل اجتماعی و اقتصادی می­دانیم برنامه­ریزی در جهت ایجاد شغل، ایجاد تفریح و شادی در جوانان و نیز آموزش مهارت­های زندگی از طریق کتب درسی می­تواند کارگشایی در زمینه کاهش استرس، کاهش پرخاشگری و به دنبال آن کاهش خشونت در قشر جوان جامعه گردد. در عین حال به نظر می­رسد ثبت موارد خشونت به تنهایی مشکلی را حل نمی­کند و می­بایست در فرم­های مربوط به ثبت حوادث ستونی برای علت ایجاد خشونت اضافه گردد و برای کاهش موارد در زمینه علل آن برنامه­ریزی­های لازم انجام شود.

Title: استرس شغلي و نوبت كاري در كاركنان شركت پلي اكريل اصفهان

http://knh.shmu.ac.ir/index.php/site/article/view/1026

مقدمه: در جوامع صنعتي نوبت كاري به­ويژه نوبت شب بسيار شايع است. نياز بدن به سازگار شدن با سيكل­هاي متفاوت خواب و بيداري منجر به استرس و اختلالات سلامتي مي­شود. اين مطالعه به­منظور تعیین شیوع استرس شغلي و بررسی ارتباط آن با شيفت كاري در بين كاركنان شركت پلي اكريل اصفهان انجام گرفت. مواد و روش­ها: يك مطالعه مقطعي در بين 227 نفراز كاركنان شركت پلی اکریل با استفاده از روش پرسش­نامه­ای خود ایفا جهت تعیین استرس شغلی، نوبت کاری و سایر اطلاعات زمینه­ای انجام شد. دو مولفه استرس شغلی (عدم تعادل تلاش- پاداش (ERI) و تعهد كاري بالا (OC)) با استفاده از پرسش­نامه سيگريت سنجیده شد. تعیین ارتباط بین استرس شغلی (ERI وOC) با شیفت کاری با به­کارگیری رگرسيون لجستيك و نرم­افزار SPSS انجام گرفت. يافته­ها: 8/82% كاركنان تاكنون حداقل یک نوبت شيفت كاري داشتند و شیوع استرس شغلی بر اساس ERI و OC به­ترتیب برابر %9/26 و %4/15 بود. پس ازکنترل متغير­هاي مخدوشگر (سن، نگرش منفي، تحصيلات و نوع قرارداد) یافته­ها نشان داد نوبت کاری پیش­بینی­کننده مؤلفه ERI استرس شغلی نیست اما شانس استرس شغلی در مقایسه با افراد دارای تحصیلات دانشگاهی، برای افراد با تحصیلات پایین 7.7 برابر (OR=7.69,95% C.I:1.458 - 40.56) و برای افراد داراي ديپلم يا تحصيلات دبيرستاني 4/2 برابر (OR=2.41,0.95% C.I: 1.14- 5.10) می­باشد. هم­چنين نسبت شانس تطبیق داده شده نشان داد که شانس مؤلفه OC استرس شغلی برای نوبت کاری شب در مقایسه با نوبت کاری روز 81% (OR=1.81,0.95% CI:0.44 - 7.41) افزایش می­یابد و با افزایش یک نمره نگرش منفی شانس استرس شغلی 16% (OR=1.16,0.95% CI:1.84 - 1.24) بیش­تر می­شود. نتيجه­گيري: در این مطالعه نوبت کاری پیش­بینی­کننده معنادار هیچکدام از مولفه­های استرس شغلی نبود اما سطح تحصیلات و نگرش منفی به­ترتیب پیش­بینی­کننده ERI و OC استرس شغلی بودند.

Title: ارتباط موقعیت اقتصادی- اجتماعی و پیامدهای بهداشتی در بيماران مبتلا به ديابت نوع دو شهرستان اروميه

http://knh.shmu.ac.ir/index.php/site/article/view/1027

مقدمه: محیط اجتماعی نقش عمده­ای در شکل­گیری عوامل خطر مهم فردی ایفا می­نماید. بر اساس مطالعات انجام شده 90 در صد از تغییرات مشاهده شده در افزایش سندرم متابولیک، میرایی و ناتوانی ناشی از دیابت و بیماری­های قلبی- عروقی نمی­تواند به عوامل خطر مرسوم نسبت داده شود. شواهد موجودحاکی از تأثیر مستقیم تعیین­کننده­های اجتماعی به­ویژه فقر در کنترل و بروز بیماری دیابت از طریق مکانیسم­های بیولوژیک، روانشناختی و رویه­های اجتماعی در طی زندگی فرد می­باشد. علاوه بر نقش مؤثر این تعیین­کننده­ها در کنترل و بروز بیماری، می­توان به نقش مؤثر محیط اجتماعی در پذیرش رفتارهای بهداشتی که کمک شایانی به کاهش بروز و کنترل مطلوب بیماری نیز اشاره نمود. مواد و روش­ها: اين مطالعه به­روش مقطعي- تحليلي، در نمونه جمعیتی 250بیمار مبتلا به دیابت نوع دو که در شش ماهه اول سال 88-89 به واحد دیابت وانجمن دیابت استان آذربایجان غربی مراجعه کرده بودند انجام شد. اطلاعات مرتبط با ویژگی­های دموگرافیک و متغیرهای سنجش وضعیت اقتصادی- اجتماعی شامل بعد خانوار، تعداد اتاق، نوع شغل زن و مرد در خانواده، سطح سواد، وضعیت مسکن و مدت ابتلا به بیماری توسط پرسش­نامه جمع­آوری گردید. اطلاعات مرتبط با نشانگرهای کنترل قند خون وابتلا به فشار خون از پرونده بیمار استخراج گردیده ونمایه توده بدنی بیمار و فشار خون بیمار در زمان مراجعه بیمار به مراکز اندازه­گیری و ثبت گردید. داده­ها با استفاده از آزمون­های تی مستقل، آنالیز واریانس یک طرفه، رگرسیون خطی و رگرسیون لاجستیک تجزیه و تحلیل شد. يافته­ها: در این پژوهش بین نمره وضعیت اقتصادی- اجتماعی و سطح کنترل شده هموگلوبین گلیکوزیله (کم­تر از 7 درصد)، فشارخون سیستولیک ودیاستولیک کنترل شده (به­ترتیب فشارخون سیستولیک زیر 140 میلی­متر جیوه و فشارخون دیاستولیک زیر 90 میلی­متر جیوه) ارتباط معناداری مشاهده شد، (مقدار عدد پی به­ترتیب 021/0، 039/0، 016/0). هم­چنین بین قند خون ناشتای بیماران و نمره وضعیت اقتصادی- اجتماعی، ارتباط معناداری مشاهده شد، (مقدار عدد پی مساوی 009/0). ولی ارتباط بین نمره وضعیت اقتصادی- اجتماعی و سطح مطلوب قند خون ناشتا (کم­تر از 130) از نظر آماری معنادار نبود. شانس ابتلا به فشارخون در بیماران دیابتی به ازای هرواحد افزایش نمره وضعیت اقتصادی- اجتماعی 2/3 درصد کاهش می­یافت، ولی این ارتباط از نظر آماری معنادار نبود، (مقدار عدد پی مساوی 182/0). نتیجه­گیری: ارتباط مشاهده شده بین نمره اقتصادی- اجتماعی و پیامدهای مهمی همچون دسترسی به سطح مطلوب قند خون، سطح مطلوب فشارخون سیستولیک ودیاستولیک بیانگر تأثیر موقعیت اقتصادی- اجتماعی درکنترل قند و فشارخون در بیماری دیابت می­باشد.

Title: مطالعه مورد شاهدی نقش برخی از عوامل اجتماعی مؤثر بر انتخاب روش سزارین در شهرستان دامغان

http://knh.shmu.ac.ir/index.php/site/article/view/1028

مقدمه: سزارین به­عنوان یک روش زایمان در تمام دنیا با اندیکاسیون­های تعریف شده، درصدی از موارد زایمان را به خود اختصاص می­دهد ولی همواره بار اقتصادی و بیماری ناشی از آن بیش­تر از زایمان طبیعی بوده است. روند فزاینده شیوع سزارین در دهه­های اخیر، توجه به تمام ابعاد پزشکی، روان شناختی و اجتماعی آن را گوشزد می­نماید. بررسی حاضر به­منظور تعیین نقش برخی از عوامل اجتماعی در انتخاب این روش به مورد اجرا در آمده است. مواد و روش­ها: یک مطالعه مورد شاهدی با استفاده از پرسش­نامه تحقیق به روش مصاحبه حضوری بر روی مادران مراجعه­کننده به مراکز بهداشتی درمانی شهری به مورد اجرا درآمد. در مجموع تعداد 120 مادر با زایمان سزارین (مورد) و 90 مادر با زایمان طبیعی (شاهد) مورد بررسی قرار گرفتند. انالیز داده­ها با نرم­افزار SPSS و با استفاده از روش­های توصیفی و تحلیلی انجام شد. يافته­ها: میانگین سنی، سطح تحصیلات، اشتغال، تعداد زایمان، شغل همسر، تعداد فرزندان و محل سکونت مادران در دو گروه اختلاف معناداری را نشان نداد. از بین عوامل اجتماعی مورد نظر، تحصیلات دیپلم و بالاتر مادر } OR=1.88(1.02-3.46}، توصیه همسر و خانواده همسر } OR=0.17(0.08-0.34} و مادر و خانواده زن } OR=0.46(0.24-0.89} ازتباط معناداری با سزارین داشته است (05/0P<). نتیجه­گیری: با توجه به این که تنها زایشگاه دامغان دولتی می­باشد، درصد بالای سزارین در آن لازم است مورد توجه قرار گیرد. نقش خانواده در توصیه به انجام زایمان طبیعی نکته قابل ملاحظه­ای می­باشد که با آموزش­های بیش­تر می­توان به گسترش زایمان طبیعی نايل آمد.

Title: اعتياد و سلامت اجتماعي

http://knh.shmu.ac.ir/index.php/site/article/view/1029

مقدمه: سوء مصرف و وابستگی به مواد مخدر، یکی از معضلات و نگرانی­های عمده جهان امروز و عامل بازدارنده رشد و شکوفایی جامعه است. پژوهش حاضر با هدف تعیین الگوی سوء مصرف مواد مخدر و فراوانی رفتارهای پرخطر در مراجعین به مراکز DIC و MMT شهرستان شاهرود انجام شد. مواد و روش­ها: اين پژوهش از نوع مطالعات توصيفي- مقطعي و كاربردي مي­باشد. در اين مطالعه پرسش­نامه­اي مشتمل بر 10 سؤال عمومي و 32 سؤال اختصاصي در خصوص اعتیاد و رفتارهای پرخطر استفاده شد. داده­ها از طریق مصاحبه و بررسی پرونده افراد تحت درمان در مراکز DIC و  MMT استخراج شد. داده­ها پس از کدگذاری، وارد نرم­افزار SPSS شده و مورد تحلیل آماری قرار گرفت. يافته­ها: میانگین سنی افراد مورد مطالعه 8/34 بود. 5/97% افراد مرد بودند. 7/92% در شهر و 3/7% ساکن روستا بودند. تدخین 4/44% طریقه مصرف مواد مخدر در افراد مورد مطالعه قبل از مراجعه به مراکز DIC و MMT بوده و پس از آن خوردن (7/25%) و تزریق (7/12) در رده­های بعدی می­باشند. کراک بیش­ترین ماده مخدر مورد استفاده در افراد مورد مطالعه قبل از مراجعه به مراکز بوده و پس از آن تریاک و شیره در رده­های بعدی قرار داشتند. در این افراد مواد زیر به­ترتیب در طی مدت زمان اعتیاد تجربه شده­اند: تریاک (313 مورد) 42/88%، شیره (232 مورد) 54/65%، کراک (170 مورد) 02/48%، الکل (107 مورد) 23/30%، حشیش (70 مورد) 77/19%، هروئین (59 مورد) 67/16% و سایر مواد (23 مورد) 50/6% بود. بین طریقه مصرف با جنس، تحصیلات، شغل و وضعیت تأهل رابطه معناداری مشاهده نگردید ولی با محل سکونت (014/0=P)، درآمد (03/0=P)، سابقه مصرف سیگار (001/0=P) رابطه معناداری وجود داشت. نتيجه­گيري: با عنایت به پایین بودن سن شروع سیگار، وجود بسیاری از رفتارهای پرخطر در افراد، وجود سابقه اعتیاد در حدود نیمی از خانواده­های افراد مورد مطالعه توجه بیش­تر به این گروه و تدوین قوانین راهبردی و افزایش مراکز ارايه خدمت و آموزش مهارت­های زندگی در مدرسه، خانواده و اجتماع می­تواند کمک شایانی به پیشگیری از عواقب ویرانگر اعتیاد نماید.

Title: نابرابری در ابتلا به عوامل خطر بيماري¬هاي غيرواگير در شهرستان شاهرود

http://knh.shmu.ac.ir/index.php/site/article/view/1036

مقدمه: بررسی شاخص نابرابری در ابتلاء به عوامل خطر بیماری­های غیر واگیر در گروه­های اقتصادی- اجتماعی مختلف شهرستان شاهرود با استفاده از داده­های مربوط به بررسي­هاي نظام مراقبت بیماری­های غیر واگیر سال 1383. مواد و روش­ها: یک مطالعه مقطعی با حجم نمونه 1000 نفر (از هر دو جنس و به تعداد برابر) که در پنج گروه سنی 24-15، 34-25، 44-35، 54-45 و 64-55 سال قرار داشتند. برای تعیین متغیر وضعیت اقتصادی-اجتماعی، از روش آناليز مؤلفه اصلي (principle component analysis) استفاده گردید و مشاهده گردید سه متغیر شغل، وضعيت سواد و تعداد اعضاي خانوار بيش­ترين سهم را در واريانس متغير­هاي دهگانه به خود اختصاص دادند (1/80 درصد). پس از ساخت متغیر جدید، هر يك از عوامل خطر بيماري­هاي غير واگير در سطوح مختلف اين متغير بررسي و شاخص­هاي نابرابري اندازه­گیری شد. یافته­ها: شاخص مرکزیت (Concentration Index) برای فشارخون، دیابت، چاقی و کلسترول بالا به ترتیب برابر 613/0، 755/0، 575/0 و 695/0 محاسبه گردید. هم­چنین این شاخص برای بی­حرکتی، مصرف سیگار، تغذیه نامناسب و حوادث به ترتیب برابر 509/0، 638/0، 511/0 و 582/0 به­دست آمد. بررسي شاخص و منحني­هاي concentration نشان­دهنده وجود نابرابري در ابتلاء به عوامل خطر بيماري­هاي غيرواگير در شهرستان شاهرود می باشد. بيش­ترين نابرابري مربوط به ديابت و با درجات کم­تر مربوط به كلسترول بالا، فشارخون بالا و چاقي است. در مورد مصرف سيگار و حوادث نابرابري واضح نیز وجود داشت اما نابرابری در بيحركتي و وضعيت تغذيه دیده نشد. نتیجه­گیری: ابتلا به عوامل خطر بيماري­هاي غيرواگير در شهرستان شاهرود در سال 83 در گروه­هاي مختلف اقتصادي- اجتماعي با نابرابری همراه بوده است. انجام مطالعات وسيعتر با حجم نمونه كافي و با استفاده از سؤالات مناسب در خصوص سنجش وضعيت اقتصادي- اجتماعي برای شناسايي نابرابري ضروري به نظر مي­رسد.

Title: بررسي كيفيت زندگي در بيماران مبتلا به پاركينسون در شيراز

http://knh.shmu.ac.ir/index.php/site/article/view/1035

مقدمه: بيماري پاركينسون يك بيماري عصبي پيشرونده مزمن مي­باشد كه حدود 1% از افراد بالاي 65 سال را درگير مي­كند و بر كيفيت زندگي بيماران تأثير مي­گذارد. و ما در اين مطالعه به بررسي كيفيت زندگي در بيماران مبتلا به پاركينسون در شيراز پرداخته­ايم. مواد و روش­ها: پژوهش حاظر يك مطالعه مقطعي و توصيفي- تحليلي مي­باشد. 108 نفر بيمار مبتلا به پاركينسون در اين مطالعه شركت داشتند. ايزار گردآوري داده­ها شامل پرسش­نامه سنجش كيفيت زندگي در بيماران مبتلا به پاركينسون (PDQL)، شاخص خواب پيتز برگ (PSQI)، مقياس شدت خستگي (FSS) و مرحله بيماري با شاخص Hohen&yahr سنجيده شد. جهت تجزيه و تحليل داده­هاي گردآوري شده نرم­افزار SPSS و آزمون­هاي من ويتني، كروسكال واليس و ضريب همبستگي اسپيرمن به­كار گرفته شد. يافته­ها: 108 بيمار (62%) 76 مرد و (38%) 41 در اين مطالعه شركت داشتند. ميانگين± انحراف معيار سن بيماران 73/11±49/64 و ميانگين ± انحراف معيار نمره كيفيت زندگي 29/62±05/121 بود و اكثريت بيماران در مرحله­هاي 2 و 3 بيماري قرار داشتند. وضعيت خستگي، كيفيت خواب و مرحله بيماري با كيفيت زندگي ارتباط معناداري نشان دادند (001/0P<) و مدت زمان ابتلا با بيماري با نمره كيفيت زندگي در ابعاد علائم سيستميك26/0R= -، 01/0P<، عملكرد اجتمايي 21/0R= - و حيطه كلي زندگي 23/0R= - 05/0P< ارتباط معكوس و معنادار نشان داد. نتيجه­گيري: بيماري پاركينسون يك بيماري مزمن و غير قابل درمان مي­باشد ولي با شناسايي عوامل كاهش­دهنده كيفيت زندگي مانند عدم كيفيت خواب، خستگي مزمن، و اقدامات مداخله­اي مثبت در اين خصوص و هم­چنين جلوگيري از پيشرفت بيماري مي­توان كيفيت زندگي اين گروه از بيماران را ارتقاء داد.

Title: The study of influencing factors over smoking among boy university students studying in Kermanshah city

http://knh.shmu.ac.ir/index.php/site/article/view/1034

Introduction: Smoking is the first preventable cause of death in the world. This problem is one of the most important difficulties of public health which has involved adolescents and young people. Since smoking incidence pattern has changed and needed to be recognized ,getting involved the community and also health officials in this field is so essential and at the same time few researches have been conducted about this matter, present study got to perform to determine influencing factors in smoking (on the basis of BASNEF Model which is a new one of health education models derived from behavioral sciences)among boy university students studying in kermanshah city. Methods: This is a descriptive-analytical and cross-sectional study in which 569 students (from Razi University, Kermanshah University Of Medical Sciences, Islamic open University, Payam noor University) studying in Kermanshah in the year 1387.Sampling method was Snowball and data collected by researcher- made questionnaire consisting of 70 questions included 5 sections which analyzed by SPSS software through Central Indices, One-Way Variance analysis and X2. Results: Knowledge and attitude grade averages about smoking among students were , in turn, 89±7/1 and 72/7±6/2 in this study. Smoked cigar number average was 10/7 ones per day. There was no any significant relation between smoked cigar numbers and university kind or educational level of students. The most frequency of enabling factors for smoking was Easy access to cigar and the most influencing social norm in smoking was students close friends. The most frequent reason to smoke stated by students were to keep themselves calm (38/3%),not to have suitable pastimes and to be so curious to try smoking for the first time. Conclusion: Having friends smoking is the most considerable factor to attract students to be caught by this habit which demonstrate the role of mate group pressure among young people. The second outstanding. Factor among social norms was having smoking parents.Parents are considered as one of the reference group for their children so that they immitate their parents behaviors during decision making. Their instructors in university and TV stars were the later ones influenced their intention to smoke.

Title: بررسی الگوی باروری سن ازدواج و استفاده از روش¬های تنظیم خانواده در بین اقوام مختلف شهرستان اهواز در سال 1388

http://knh.shmu.ac.ir/index.php/site/article/view/1033

مقدمه: قوميت به­عنوان زمينه­اي كه رفتار باروري در آن شكل مي­گيرد، جايگاه خاص و اساسي درمطالعات باروري در جوامع چند قوميتي دارد. با اين حال، علي­رغم ساختار چند قوميتي ايران هنوز تحقيقات اندكي در زمينه ارتباط قوميت و باروري صورت گرفته است. هدف از پژوهش حاضر، بررسی الگوی باروری، سن ازدواج و استفاده از روش­های تنظیم خانواده در بین اقوام مختلف شهرستان اهواز بود. مواد و روش­ها: طرح تحقیق در این پژوهش، پيمايشي، و تکنیک مورد استفاده برای جمع­آوری اطلاعات پرسش­نامه محقق ساخته بود. جهت تجزیه و تحلیل داده­ها از روش آمار توصيفي و آزمون كاي دو و رگرسيون لجستيك با به­كارگيري نرم­افزار SPSS استفاده شد. نمونه شامل 783 نفر از زنان ازدواج کرده مراجعه­کننده به مراکز بهداشتی درمانی شهرستان اهواز بودند که به­صورت تصادفی از میان کلیه مراجعه­کنندگان زن به مراکز بهداشتی درمانی انتخاب شدند يافته­ها: یافته های پژوهش نشان دادند که بین الگوی باروری و سن ازدواج و استفاده از روش­های تنظیم خانواده در بین اقوام مختلف در سطح معناداری (05/0P<) رابطه معناداری وجود دارد. نتيجه­گيري: نتایج این مطالعه نشان داد که سطح سواد زنان یک عامل کاملاً تأثیرگذار می­باشد به­طوری که پس از وارد شدن به مدل رگرسیون و قرار گرفتن در کنار سایر عواملی مانند قومیت، سن ازدواج و روش­هاي تنظیم خانواده کماکان اثر خود را حفظ نمود. عامل قومیت در کنار سایر عوامل مؤثر بر باروری نظیر سن ازدواج و سواد زن قرار می­گیرد ارتباط معناداری با باروری ندارد. بنابراین لازم است در برنامه­های توسعه کشوری مقوله قومیت­ها گنجانده شود زیرا نمی­توان یک برنامه و سیاست جمعیتی خاص را برای تمام گروه­های قومی اتخاذ و اجرا نمود لزوم توجه به قومیت­ها و رفتار جمعیتی خاص در سیاستگذاری­ها اجتناب­ناپذیر می­باشد.

Title: بررسي عوامل مؤثر بر بهبود كيفيت درک شده مراقبت¬های دوران بارداری بر اساس مدل SERVQUAL

http://knh.shmu.ac.ir/index.php/site/article/view/1032

مقدمه: اولين گام در بهبود كيفيت خدمات، سنجش كيفيت خدمات مي­باشد. و براي اين منظور مدل­هاي زيادي ارايه شده است، كه رايج­ترين آن­ها  SERVQUALاست. اين مدل توسط پاراسورامان و همكارانش در سال 1985 ارايه شد. و شامل پنج بعد است. بر اين اساس اين تحقيق براي بررسي عوامل مؤثر بر بهبود كيفيت درك شده مراقبت­هاي پيش از زايمان زنان باردار روستايي شهرستان گچساران در بهار 1387 بر اساس مدل SERVQUAL صورت گرفت. مواد و روش­ها: در مطالعه توصيفي تحليلي حاضر كه به روش مقطعي در بهار 1387 انجام شد كليه زنان باردار روستايي شهرستان گچساران كه دراين مقطع داراي پرونده بارداري در خانه بهداشت بوده، حداقل سه ماه از خدمات خانه بهداشت استفاده كرده بودند و حداقل 6 ماه دريكي از روستاهاي تحت پوشش خانه بهداشت سكونت داشته­اند مورد پرسش قرار گرفتند. گردآوري داده­ها به­وسيله پرسش­نامه­اي شامل دو بخش، بخش اول اطلاعات زمينه­اي و بخش دوم پرسش­نامه­اي دو قسمتي شامل ادراكات و انتظارات تهيه شده است. براي بررسي روايي پرسش­نامه ازنظر متخصصين استفاده شده است، و براي پايايي از آزمون مجدد استفاده شده است براي تجزيه و تحليل داده­ها از نرم­افزار SPSS استفاده گرديد. يافته­ها: اين بررسي نشان داد كه در تمام ابعاد كيفيت خدمات بين ادراكات و انتظارات زنان باردار روستايي شكاف وجود دارد. به عبارتي انتظارات آنان از خدمات بيش­تر از چيزي است كه در عمل دريافت مي­كنند و بيش­ترين شكاف در بعد عوامل محسوس و كم­ترين در بعد پاسخگويي است. نتيجه­گيري: بنابراين مديران دانشگاه علوم پزشكي ياسوج و شبكه بهداشت و درمان گچساران فرصت­هاي زيادي براي ارتقاء كيفيت خدمات در خانه­هاي بهداشت دارند و براي ارتقاء كييفيت خدمات لازم است نسبت به تجهيز خانه­هاي بهداشت اقدام كنند، و به كاركنان خانه­هاي بهداشت براي برقراري ارتباط عاطفي با زنان باردار و ارتقاء مهارت­هاي فني، آموزش­هاي مستمر ارايه دهند.

Title: بررسی ميزان خشونت خانگي و ارتباط آن با افسردگي در زنان

http://knh.shmu.ac.ir/index.php/site/article/view/1031

مقدمه: خشونت خانگی يک اپيدمی رو به رشد جهانی است که می­تواند منجر به پيامدهای جسمی و روانی فراوانی شود، لذا اين مطالعه به­منظور تعيين ميزان خشونت خانگي و ارتباط آن با افسردگي در زنان مراجعه­کننده به واحد بهداشت خانواده مراکز بهداشتي درماني شهر تهران طراحی و اجرا شد. مواد و روش­ها: اين پژوهش يک مطالعه مقطعی می­باشد که تعداد 811 نفر از زنان به روش نمونه­گيری طبقه­ای تصادفی انتخاب شدند. ابزار گردآوری اطلاعات پرسش­نامه بود که از طريق مصاحبه تکميل گرديد، برای تجزيه و تحليل داده­ها از آمار توصيفی و استنباطی (آزمون آماری کای دو، ضريب همبستگی پيرسون) استفاده شد. يافته­ها: ميزان خشونت خانگی و افسردگی به­ترتيب 3/88% (خشونت جسمی 4/25%، خشونت خشونت عاطفـی 3/87% و خشونت جنسی1/39%) و 1/37% (افسردگی خفيف 7/28%، افسردگی متوسط 2/8% و افسردگی شديد 2/0%) بود. آزمون آماری کای دو ارتباط معناداری ميان هر سه نوع خشونت با وضعيت افسردگی (افسرده و غير افسرده) نشان داد، در مقايسه با گروه خشونت نديده نسبت شانس وقوع افسردگی در کسانی که خشونت عاطفی، خشونت جسمی، خشونت جنسی و خشونت را در طی سال گذشته تجربه کرده بودند به­ترتيب (344/5-85/1) CI144/3، (284/4-232/2) CI 092/3، (214/4-326/2) CI 132/3 و (281/6-003/2) CI 547/3 [با فاصله اطمينان 95%] بود. نتيجه­گيری: با توجه به شيوع بالای خشونت خانگی و افسردگی و وجود ارتباط بين آن­ها، غربالگری روتين زنان از لحاظ مواجهه با خشونت خانگی و هم­چنين افسردگی از طريق برنامه­ريزی­های مناسب توصيه می­شود.

Title: بررسی اپیدمیولوژیکی اختلالات جنسی در بیماران قلبی- عروقی نسبت به افراد سالم مشابه در سال 88 شهرستان دامغان

http://knh.shmu.ac.ir/index.php/site/article/view/1030

مقدمه: بیماران قلبی و عروقی نیز مانند بسیاری از بیماری­های دیگر در معرض خطر عوارض بیماری و عوارض داروهای مصرفی به­دلیل بیماری در خصوص اختلالات جنسی هستند و با توجه به شیوع بسیار بالای بیماری در کشور اختلالات جنسی هم در این افراد و جامعه در حال افزایش است که ضرورت دارد با توجه به شناخت وضعیت موجود درمان و سطح مختلف پیشگیری اقدام نمود. مواد و روش­ها: نوع مطالعه توصیفی- تحلیلی است و جامعه مورد هدف 200نفر از هرگروه (ابتلایان به بیماری­های قلبی و عروقی و افراد سالم) می­باشند و روش جمع­آوری امار با پرسش­نامه محقق ساخته از گروه­های هدف که به مرکز مشاوره سایا مراجعه نموده­اند انتخاب گردیده­اند و توسط خود محقق پرسش­نامه تکمیل و مشاهدات ثبت شده است و تجزیه و تحلیل اطلاعات با استفاده از نرم­افزار EPI انجام گرفته است. شناخت میزان­های بروز و شیوع اختلالات جنسی در مبتلایان به بیماران قلبی و عروقی، مصرف­کنندگان داروهای قلبی و عروقی و افراد سالم. یافته­ها: یافته­ها بیانگر این موضوع است که بیماران قلبی و عروقی 26 برابر افراد بیمار قلبی و عروقی که داروهای قلبی مصرف می­نمایند 5 برابر بیش­تر از افراد به ظاهر سالم دچار اختلالات جنسی مختلف بوده­اند، هم­چنین مصرف قرص­هایی مانند سید فاصیل، تاداگرا، ویاگرا و... در بیماران قلبی تا 70 درصد و هفت برابر افراد سالم می­باشند هم­چنین در نتایج کیفیت ارتباط جنسی دو گروه شامل ارگاسم- رضایت­مندی جنسی، توانائی در لذت بردن، مشکلات نعوظ واژینیسم و... اندازه­گیری می­گردید. نتیجه­گیری: با توجه به نتایج حاصله و فراوانی­ها در بیماران قلبی و عروقی اختلالات جنسی بسیار بالاتر از افراد سالم است برای پیشگیری از پیامدهای نتایج ضروری است آموزش­های بهداشت جنسی در زمان مراجعه به پزشک جهت درمان و گرفتن دارو داده شود چون بیش­تر از 80 درصد اختلالات با آموزش بهداشت جنسی برطرف و یا حداقل کاهش می­یابد.

Title: بررسي وضعيت دسترسي جمعيت حاشيه¬نشين به خدمات بهداشتي درماني در شهر شيراز

http://knh.shmu.ac.ir/index.php/site/article/view/1037

مقدمه: حاشيه­نشيني نوعي سكونت غير رسمي به­صورت نا متعارف در حاشيه شهرها است كه از كم­ترين امكانات رفاهي و خدماتي از جمله خدمات بهداشتي برخوردارند. هدف اين مطالعه بررسي وضعيت دسترسي اين افراد به خدمات بهداشتي و درماني مي­باشد. مواد و روش­ها: جمعيت هدف در اين مطالعه كليه خانوارهاي حاشيه­نشين شهر شيراز بود كه از اين خانوارها يك نمونه 380 تايي به روش نمونه­گيري طبقه­بندي انتخاب گرديد. اطلاعات در فرم مخصوص شامل اطلاعات دموگرافيك و نحوه دريافت خدمات بهداشتي جمع‌آوري شد. داده­ها با استفاده ازروش­هاي آماري توصيفي تجزيه و تحليل گرديد. يافته­ها: نتايج نشان داد كه بعد خانوار در مناطق مذكور 5 است. از 380 خانوار مورد بررسي 75% (285 خانوار) از خانوارها دسترسي فيزيكي به مراكز بهداشتي درماني دولتي را بين 10 تا30 دقيقه و بقيه اين فاصله را بيش از 30 دقيقه عنوان كرده‌اند. ميزان پوشش روش­هاي پيشگيري از بارداري 4/46% (51%-41%CI: 95%) برآورد شد. پوشش واكسیناسيون كودكان 98% (99%-97%CI: 95%) و پوشش واكسن هپاتيت جوانان 50% (55%-45%CI:95%) بود. نتيجه­گيري: میزان عدم دسترسی به خدمات بهداشتی و درمانی خانوارهای حاشيه­نشيان شهر شيراز (25%) در حد قابل قبولی نبود. هم­چنين استفاده و پوشش خدماتی هم چون تنظيم خانواده آنان در مقايسه با متوسط پوشش تنظيم خانواده روستاهاي استان (81/67%) نيز پايين­تر است (001/0P<). بنابراین علاوه بر دسترسی به خدمات، استفاده و هم­چنین ارایه خدمات به گروه هدف نیز حایز اهمیت است.
